# Hexafluoroisopropyl alcohol mediated synthesis of 2,3-dihydro-4H-pyrido[1,2-a]pyrimidin-4-ones

**DOI:** 10.1038/srep36316

**Published:** 2016-11-02

**Authors:** Mohammad A. Alam, Zakeyah Alsharif, Hessa Alkhattabi, Derika Jones, Evan Delancey, Adam Gottsponer, Tianhong Yang

**Affiliations:** 1 Department of Chemistry and Physics, College of Science and Mathematics, Arkansas State University, Jonesboro, AR, 72467, USA; 2 Molecular Biosciences Graduate Program, Arkansas State University, Jonesboro, AR 72467, USA

## Abstract

An efficient synthesis of novel 2,3-dihydro-4H-pyrido[1,2-a]pyrimidin-4-ones has been reported. Inexpensive and readily available substrates, environmentally benign reaction condition, and product formation up to quantitative yield are the key features of this methodology. Products are formed by the aza-Michael addition followed by intramolecular acyl substitution in a domino process. The polar nature and strong hydrogen bond donor capability of 1,1,1,3,3,3-hexafluoropropan-2-ol is pivotal in this cascade protocol.

The 1,3-nitrogenous bicyclic frameworks have been illustrious in drug discovery. Many pyrido-pyrimidinone scaffolds (i.e., Fig. 1) have occupied privileged position in medicinal chemistry due to their unprecedented biological activities. It is a key constituent of numerous natural products possessing wide range of therapeutic properties including antitumor, anti-influenza, oxidative burst inhibition, lipid droplet synthesis inhibition, and anti-obesity properties^1^,^2^,^3^,^4^,^5^,^6^,^7^,^8^,^9^,^10^. Recent studies have revealed that the molecules bearing pyrido-pyrimidinones are in different phases of drug development to treat cancer, hypertension, neurological disorders, etc^11^,^12^,^13^,^14^,^15^,^16^. Some of them have been recognized as aldose reductase inhibitors, efflux pump inhibitors, and hepatitis C virus NS3 protease inhibitors. 1,3-Nitrogenous bicyclics also include marketed drugs for depression and asthma treatment (Fig. 2)^2^,^6^,^9^,^14^,^15^.

Synthesis of structural variant of pyrido-pyrimidinones is challenging. Zeng *et al*. have utilized palladium catalyzed C-H activation entailed carbonylative cycloamidation of ketoimines (Fig. 3, Case A)^17^. It requires a bimetallic combination of palladium and copper along with toxic carbon monoxide in a sophisticated reaction setup. Spring and coworkers have used 2-aminopyrimidine and alkynoates in bicyclic pyrimidones synthesis. The use of butyl lithium is critical and require continuous monitoring of anhydrous condition (Fig. 3, Case B)^18^. Bicyclic pyrimidones have also been synthesized by using *β*-oxo esters and 2-amnionpyrimidines in the presence of BiCl_3_ catalyst (Fig. 3, Case C)^11^. The use of alkyne Michael addition helps in shifting the position of carbonyl group in pyrimidones^19^. Dai *et al*. have reported the Michael addition of anilines with acrylates by using polymer-supported AlCl_3_ in which they reported one cyclized molecule; 2,3-dihydro-4H-pyrido[1,2-a]pyrimidin-4-one (**1**) among other aza-Michael adducts^20^. From literature survey it was apparent that there are no reports on the dihydropyrido-pyrimidinones chemistry and their biological activity. It motivated us to design and developed a protocol for their synthesis and investigate their biological activities (Fig. 3, Case D). In this article, we present our results involving sustainable design and catalyst free synthesis of 2,3-dihydro-4H-pyrido[1,2-a]pyrimidin-4-one derivatives.

## Results and Discussion

**Figure 4:**
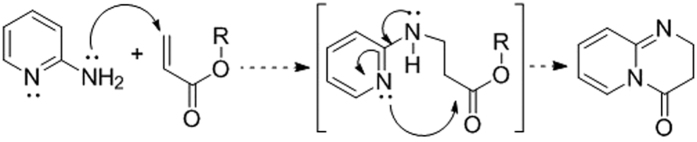
Proposed reaction pathway towards pyrimidones.

Engage in small molecule research, we wanted to explore the synthesis of dihydropyrido-pyrimidinones and their biological activities. Dearth of literature and our interest in its medicinal chemistry encouraged us to design and synthesize bicyclic dihydropyrido-pyrimidinone derivatives. We speculated that the introduction of amine group at second position in pyridine will increase the nucleophilicity of ring-nitrogen and activity of amino group towards nucleophilic aza-Michael addition to electron deficient double bonds. Accordingly, a reaction pathway was envisaged where the amine group undergoes aza-Michael addition and ring nitrogen participates in the exo-trig cyclization for the formation of bicyclic pyrimidinones architecture (Fig. 4).

**Figure 5:**
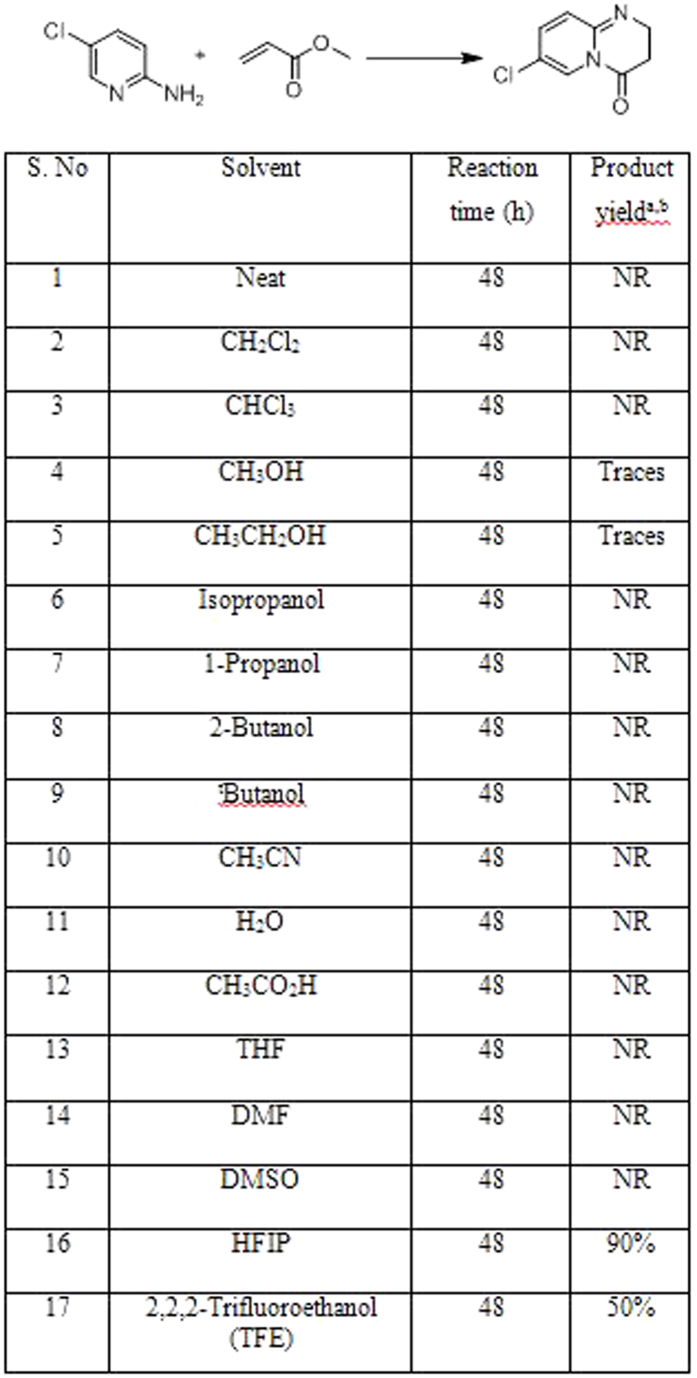
Solvent screening and reaction optimization.

The first step in the synthesis of dihydropyrido-pyrimidinones is the aza-Michael addition of 2-aminopyridines with *α,β*-unsaturated ester. The addition of aromatic amine over electron deficient double bond is well known. In most of the cases, it requires acid catalyst to facilitate nucleophilic aza-Michael type addition. We believed that the use of aromatic nitrogen ring in conjugation with amine functional group increase the probability of catalysis free aza-Michael type addition; which on intramolecular cyclization may yield the desire product. Our aim was to find the reaction condition and appropriate solvent for the facile conversion of aminopyridine to desired dihydropyrimido derivatives. We began our study with the reaction of 2-amino-5-chloropyridine and methyl acrylate in different solvents towards the aza-Michael addition and cyclization protocol. In the beginning the reaction outcome was disappointing as it did not yield the desire product in most of the solvents (Fig. 5, entry 1–15). The increase in reaction temperature did not alter the reaction outcome. The twilight of success started emerging with the reaction in methanol and ethanol (Fig. 5, entries 5 and 6) as it gave the desired product in detectable amount. We attributed the formation of dihydropyrido-pyrimidinones to high polarity and inter molecular hydrogen bonding with polar hydroxyl group of methanol and ethanol. Hitherto, we explored the reaction in fluorinated alcohols (Fig. 5, entries 16 and 17). The reaction in hexafluoroisopropanol (HFIP) persuaded the aza-Michael addition cyclization with the quantitative yield of desired product (Fig. 5, entry 16). The most promising part of the reaction was the purity of the isolated product after evaporation or filtration.

**Figure 6:**
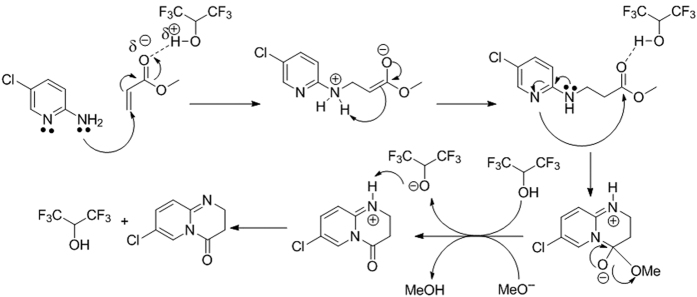
Tentative mechanism for the dihydropyrido-pyrimidinones formation.

The aza-Michael addition is greatly facilitated by the strong inter molecular hydrogen bonding between hexafluoro-2-propanol (HFIP) and carbonyl group of the Michael acceptor. After the first step, the nucleophilicity of ring nitrogen increases exponentially due to the direct conjugation with amino group; facilitating the exo-trig cyclization to form thermodynamically stable dihydropyrido-pyrimidinones (Fig. 6).

**Figure 7:**
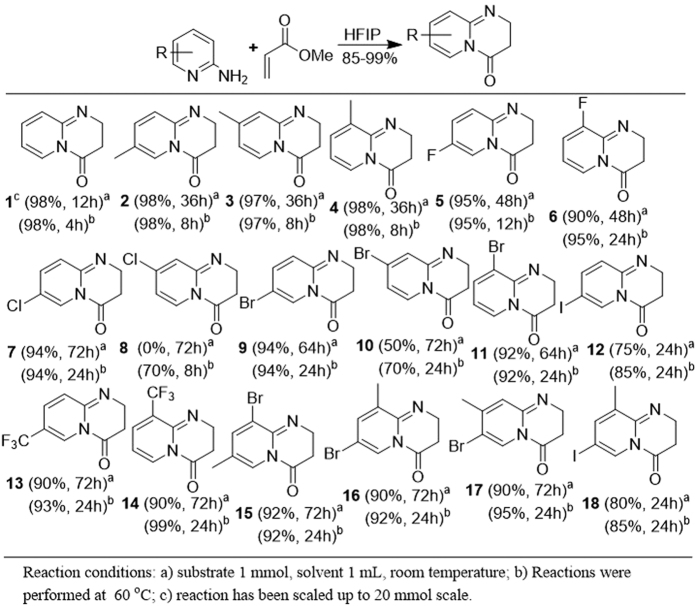
Synthesis of dihydropyrido-pyrimidinones using aza-Michael-cyclization strategy.

The reaction outcome impelled us to use HFIP as a reaction medium. Subsequently, reactions were performed in HFIP at room temperature without the use of external catalyst. A wide range of substituted 2-aminopyridines were treated with an *α,β*-unsaturated ester. The reaction of 2-aminopyridine with methyl acrylate (Michael acceptor) completed in 12 hours to give the product (**1**) in quantitative yield. The presence of electron donating or electron withdrawing substituent had an unfavorable response on the rate of reaction. It may be due to an imbalance on the optimum electron density in the aromatic system (Fig. 7, Substrate **2–16**). The methyl substituted 2-aminopyridines formed dihydropyrido-pyrimidinones without differentiating the position of methyl substituent in the ring system. As the use of 2-amino-3-methylpyridine, 2-amino-4-methylpyridine, and 2-amino-5-methylpyridine in catalyst free cascade aza-Michael addition cyclization protocol does not alter reaction rate and product outcome. All methyl substituted substrates gave the desired dihydropyrido-pyrimidinones (**2**, **3**, and **4**) in very high yield after 36 hours of stirring at room temperature. The halogen substituted 2-aminopyridines were also utilized in the synthesis of dihydropyrido-pyrimidinones. Fluoro substituted 2-aminopyridine gave the desired products (**5** and **6**) in excellent yield and required 48 hours for the completion of the reaction. Refluxing 2-amino-4-chloropyridine in HFIP for ~8 h formed the product (**8**) in 70% yield further refluxing of the reaction to completely consume the reactants resulted in the hydrolysis of the pyrido-pyrimidinone to acid derivative. Reactions of bromo substituted aminopyridines were found to be proceeding faster, as these reactions were completed in 48 hours at ambient conditions to give the desired products (**9, 10**, and **11**). The iodo derived substrate also gave the cyclized product (**12**) in 85% yields. The trifluoromethyl substituted 2-aminopyridines too gave the dihydropyrido-pyrimidinones (**13** and **14**) in excellent yield. The disubstituted 2-aminopyridines were also investigated in the cascade aza-Michael cyclization strategy. Interestingly, all the substrates gave the desired products (**15**, **16**, **17**, and **18**) in excellent yields (Fig. 7).

The effect of alkoxy group in the cyclization step was examined by treating 2-aminopyridine with methyl acrylate, ethyl acrylate, and tert-butyl acrylate. The reaction outcome confirmed that these groups does not involve in the rate determining step and have a trifling effect over the rate of the reaction. Additionally, the scaling up of the reaction to multi-gram scale does not require any special modification and product is isolated in pure form without any difficulty. The reaction solvent was easily recovered, and recycled by simple distillation to achieve the best possible Environmental Factor (E) = 0^21^.

**Figure 8:**
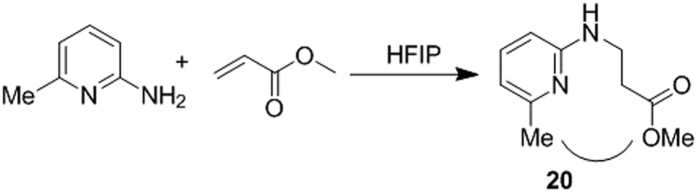
Reaction of 2-amino-6-methylpyridine and steric hindrance.

Electron withdrawing groups, specially –NO_2_, and –COOH, inhibited the formation of desired products. The analysis of reaction mixture confirms the absence of aza-Michael adducts, corroborating that fact that the electron withdrawing groups diminish the nucleophilicity of amino group, which does not allow it to participate in the aza-Michael addition. The incorporation of substituent at 6-position in 2-aminopyridine inhabited the cyclization step as the aza-Michael adduct (**19**) was isolated as a sole product in the reaction of 2-amino-6-methylpyridines with methyl acrylates (Fig. 8). The ceasing of cyclization is due to the steric hindrance created by methyl substituent at the sixth position (Supporting Information).

**Figure 9:**
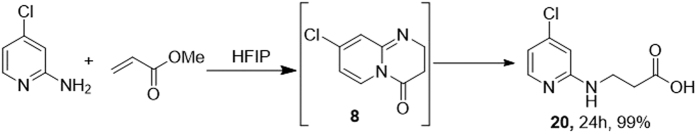
Formation of acid derivative (20) of 4-chloro-2-aminpyridine.

We found abnormal product formation for the reaction of 4-chloro-2-aminopyridine with methyl acrylate under the optimized reaction condition. Analysis of the reaction mixture showed the formation of desired product (**8**) but refluxing for longer period of time (~24 h) to complete the reaction resulted the further hydrolysis to acid derivative (**20**). The acid product (**20**) formed in quantitative yield. Abnormal product formation may be due to the strong electron withdrawing effect of chlorine at 4^th^ position, which facilitate the hydrolysis of pyrimidinone derivative (**8**) to thermodynamically stable acid derivative (Fig. 9).

**Figure 10:**
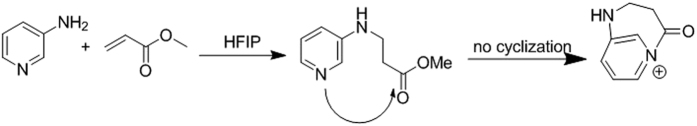
Reaction with 3-aminopyridine with methyl acrylate.

The reaction of 3-aminopyridine with methyl acrylate gave the aza-Michael addition product (Fig. 10). The exclusive formation of Michael adduct with 3-aminopyridine is may be due to increased ring strain and imbalance electronic behavior in seven member ring system.

In this article, we designed and developed a strategy for the synthesis of 2,3-dihydro-4H-pyrido[1,2-a]pyrimidin-4-one and their derivatives. These molecules are not known in literature except compound (**1**). The key factor in the discovery is the use of polar and strong hydrogen bonding hexafluoroisopropanol (HFIP) as a solvent. The use of HFIP facilitates the efficient aza-Michael addition and cyclization in one pot reaction. Detail reaction analysis supported by control experiments gave insight view of the reaction mechanism and its future scope. Ease in synthesis and scalability to multi-gram scale have been the strength of the developed methodology. It gives immense scope to generate a library of new scaffolds required for the drug discovery and biological study. The developed reaction is clean and product could be isolated by simple filtration or evaporation. Additionally, it does not require any purification as the reaction is clean and free from the unwanted by products.

## Methods

### General procedure for the synthesis of dihydropyrido-pyrimidinone

Mixture of 2-aminopyridine (1 mmol) and methyl acrylate (1.1 mmol) in 1 mL HFIP was stirred or refluxed for a specified time. Progress of the reaction was monitored by TLC. After the completion of the reaction, solid was filtered to get the product. For a homogenous reaction mixture, solvent was evaporated to get the product.

### 7-Methyl-2,3-dihydro-4*H*-pyrido[1,2-*a*]pyrimidin-4-one (2)

White solid powder, m. p. = 121.3 °C. IR (thin film, cm^−1^): 1635, 1584, 1387. ^1^H NMR (300 MHz, CDCl_3_): δ 7.39 (dd, J = 2.0, 9.0 Hz, 1 H), 7.28 (s, 1 H), 6.93 (d, J = 9.0 Hz, 1), 4.25 (t, J = 7.3 Hz, 2 H), 2.71 (t, 7.5 Hz, 2 H), 2.21 (s, 3 H); ^13^C NMR (75 MHz, DMSO-d_6_): δ = 174.9, 156.9, 142.4, 135.2, 123.3, 121.8, 50.5, 29.2, 17.2. ESI Mass (m/z): calcd for C_9_H_11_N_2_O [M + H]^+^ 163.1, found 163.1.

### 8-Methyl-2,3-dihydro-4H-pyrido[1,2-a]pyrimidin-4-one (3)

Colorless solid needles, m. p. = 145.8 °C. IR (thin film, cm^−1^): 1642, 1475. ^1^H NMR (300 MHz, CDCl_3_): δ 7.27 (d. J = 6.6 Hz, 1 H), 6.53 (s, 1 H), 6.32 (d, J = 6.5 Hz, 1 H), 4.10 (t, J = 7.1 Hz, 2 H), 2.48 (t, J = 7.3 Hz, 2 H), 2.11 (s, 3 H); 13C NMR (75 MHz, DMSO-d6): δ = 174.0, 156.5, 152.0, 136.3, 120.4, 113.8, 49.0, 28.5, 20.6. ESI Mass (m/z): calcd for C_9_H_11_N_2_O [M + H]^+^ 163.1, found 163.1.

### 9-Methyl-2,3-dihydro-4*H*-pyrido[1,2-*a*]pyrimidin-4-one (4)

Fluffy white solid, m. p. = 122.7 °C. IR (thin film, cm^−1^): 1638, 1593, 1531. ^1^H NMR (300 MHz, DMSO-d_6_ + CDCl_3_): δ 7.30 (d, J = 7.0 Hz, 1 H), 7.23 (d, J = 6.4 Hz, 1 H), 6.44 (t, J = 6.8 Hz, 1 H), 4.18 (t, J = 7.2 Hz, 2 H), 2.57 (t, J = 7.3 Hz, 2 H), 2.15 (s, 3 H); ^13^C NMR (75 MHz, DMSO-d_6_): δ = 174.9, 157.2, 138.3, 135.2, 131.7, 111.4, 50.6, 29.2, 18.3. ESI Mass (m/z): calcd for C_9_H_11_N_2_O [M + H]^+^ 163.1, found 163.1.

### 7-Fluoro-2,3-dihydro-4*H*-pyrido[1,2-*a*]pyrimidin-4-one (5)

White solid powder, m. p. = 262.0 °C. IR (thin film, cm^−1^): 1653, 1558, 1498, 1308. ^1^H NMR (300 MHz, DMSO-d_6_ + CDCl_3_): δ 8.12 (t, J = 3.7 Hz, 1 H), 7.73 (dt, J = 2.9, 7.2 Hz, 1 H), 6.67 (dd, J = 5.4 Hz, 1 H), 4.27 (t, J = 7.4 Hz, 2 H), 2.49 (t, J = 7.5 Hz, 2 H); ^13^C NMR (75 MHz, DMSO-d_6_ + CDCl_3_): δ = 174.4, 156.0, 150.5 (^1^J_C-F_ = 232.4 Hz), 131.7 (^2^J_C-F_ = 22.3 Hz), 126.3 (^2^J_C-F_ = 37.7 Hz), 123.4 (^3^J_C-F_ = 6.9 Hz), 50.0, 29.1. ESI Mass (m/z): calcd for C_8_H_8_FN_2_O [M + H]^+^ 167.1, found 167.1.

### 9-Fluoro-2,3-dihydro-4*H*-pyrido[1,2-*a*]pyrimidin-4-one (6)

White amorphous solid, m. p. = 205.0 °C. IR (thin film, cm^−1^): 1635, 1620, 1483, 1340. ^1^H NMR (300 MHz, DMSO-d_6_ + CDCl_3_): δ 7.69 (d, J = 6.5 Hz, 1 H), 7.56 (t, J = 9.4 Hz, 1 H), 6.63 (dd, J = 6.6 Hz, 1 H), 4.36 (t, J = 7.2 Hz, 2 H), 2.52–2.49 (m, 2 H); ^13^C NMR (75 MHz, DMSO-d_6_ + CDCl_3_): δ = 174.2, 149.8 (^1^J_C-F_ = 226.8 Hz), 135.6, 122.7 (^2^J_C-F_ = 17.4 Hz), 122.6, 109.3, 49.9, 29.4. ESI Mass (m/z): calcd for C_8_H_8_FN_2_O [M + H]^+^ 167.1, found 167.1.

### 7-Chloro-2,3-dihydro-4H-pyrido[1,2-a]pyrimidin-4-one (7)

White amorphous solid, m. p. = 127.0 °C. IR (thin film, cm^−1^): 1623, 1485, 1395, 826. ^1^H NMR (300 MHz, CDCl_3_): δ 7.46 (d, J = 2.3 Hz, 1 H), 7.42 (s, 1 H), 6.93 (dd, J = 1.1, 8.9 Hz, 1 H), 4.29 (t, J = 7.2 Hz, 2 H), 2.75 (t, J = 4.2 Hz, 2 H); ^13^C NMR (75 MHz, CDCl_3_): δ = 174.6, 156.7, 140.4, 135.0, 124.7, 118.4, 50.7, 28.9. ESI Mass (m/z): calcd for C_8_H_7_ClN_2_O [M + H]^+^ 183.0, found 183.0.

### 8-chloro-2,3-dihydro-4*H*-pyrido[1,2-*a*]pyrimidin-4-one (8)

White solid powder, m. p. = 142.0 °C. IR (thin film, cm^−1^): 1639, 1490, 750. ^1^H NMR (300 MHz, DMSO-d_6_): δ 8.69 (d, J = 6.8 Hz, 1 H), 7.23 (s, 1 H), 7.71 (d, J = 6.8 Hz, 1 H), 4.77 (t, J = 6.9 Hz, 2 H), 2.95 (t, J = 6.9 Hz, 2 H); ^13^C NMR (75 MHz, DMSO-d_6_): δ = 167.2, 151.0, 150.3, 143.4, 119.6, 115.0, 50.4, 28.8. ESI Mass (m/z): calcd for C_8_H_7_ClN_2_O [M + H]^+^ 183.0, found 183.0.

### 7-Bromo-2,3-dihydro-4*H*-pyrido[1,2-*a*]pyrimidin-4-one (9)

Brownish solid, m. p. = 167 °C. IR (thin film, cm^−1^): 1640, 1493, 529. ^1^H NMR (300 MHz, DMSO-d_6_ + CDCl_3_): δ 7.93 (d, J = 2.0 Hz, 1 H), 7.54 (dd, J = 2.2, 9.4 Hz, 1 H), 6.71 (d, J = 9.4 Hz, 1 H), 4.29 (t, J = 9.4 Hz, 2 H), 2.54 (t, J = 7.5 Hz, 2 H); ^13^C NMR (75 MHz, DMSO-d_6_ + CDCl_3_): δ = 174.7, 156.5, 142.8, 138.7, 123.8, 103.8, 50.0, 29.0. HRMS (ESI-FTMS Mass (m/z): calcd for C_8_H_7_BrN_2_O [M + H]^+^ 228.9820, found 228.9795.

### 8-bromo-2,3-dihydro-4*H*-pyrido[1,2-*a*]pyrimidin-4-one (10)

White solid powder, m. p. = 305.0 °C. IR (thin film, cm^−1^): 1636, 1479, 549. ^1^H NMR (300 MHz, DMSO-d_6_ + CDCl_3_): δ 8.51 (d, J = 6.9 Hz, 1 H), 7.85 (d, J = 6.8 Hz, 1 H), 7.52 (s, 1 H), 4.71 (t, J = 6.6 Hz, 2 H), 2.96 (t, J = 6.9 Hz, 2 H); ^13^C NMR (75 MHz, DMSO-d_6_ + CDCl_3_): δ = 167.4, 149.3, 142.9, 141.4, 122.8, 118.0, 50.5, 28.7. HRMS (ESI-FTMS Mass (m/z): calcd for C_8_H_7_BrN_2_O [M + H]^+^ 228.9820, found 228.9795.

### 9-Bromo-2,3-dihydro-4*H*-pyrido[1,2-*a*]pyrimidin-4-one (11)

White solid powder, m. p. = 235.0 °C. IR (thin film, cm^−1^): 1640, 1479, 541. ^1^H NMR (300 MHz, DMSO-d_6_ + CDCl_3_): δ 8.19 (d, J = 5.6 Hz, 1 H), 8.05 (d, J = 7.5 Hz, 1 H), 6.58 (t, J = 7.4 Hz, 1 H), 4.34 (t, J = 7.3 Hz, 2 H), 2.48 (t, J = 7.7 Hz, 2 H); ^13^C NMR (75 MHz, DMSO-d_6_ + CDCl_3_): δ = 174.6, 154.6, 143.3, 139.7, 116.0, 111.0, 50.7, 29.4. HRMS (ESI-FTMS Mass (m/z): calcd for C_8_H_8_BrN_2_O [M + H]^+^ 228.9820, found 228.9800.

### 7-Iodo-2,3-dihydro-4*H*-pyrido[1,2-*a*]pyrimidin-4-one (12)

Brownish solid, m. p. = 156.0 °C. ^1^H NMR (300 MHz, DMSO-d_6_): δ 8.35 (s, 1 H), 7.77 (dd, J = 2.0, 9.2 Hz, 1 H), 6.56 (d, J = 9.2 Hz, 1 H), 4.27 (t, J = 7.5 Hz, 2 H), 2.43 (t, J = 6.9 Hz, 2 H); ^13^C NMR (75 MHz, DMSO-d_6_): δ = 174.6, 153.3, 147.4, 144.1, 123.5, 49.5, 29.3. ESI Mass (m/z): calcd for C_8_H_7_IN_2_O [M] 274.9681, found 274.9678.

### 7-(Trifluoromethyl)-2,3-dihydro-4*H*-pyrido[1,2-*a*]pyrimidin-4-one (13)

Colorless solid, m. p. = 126.0 °C. IR (thin film, cm^−1^): 1652, 1558, 1336. ^1^H NMR (300 MHz, DMSO-d_6_ + CDCl_3_): δ 8.44 (s, 1 H), 7.79 (dd, J = 2.1, 9.3 Hz, 1 H), 6.82 (d, J = 9.3 Hz, 1 H), 4.37 (t, J = 7.3 Hz, 2 H), 2.52 (t, J = 7.4 Hz, 2 H); ^13^C NMR (75 MHz, DMSO-d_6_ + CDCl_3_): δ = 175.1, 157.9, 139.5, 139.4, 135.3, 122.9, 49.8, 29.1. ESI Mass (m/z): calcd for C_9_H_8_F_3_N_2_O [M + H]^+^ 217.0, found 217.0.

### 9-(trifluoromethyl)-2,3-dihydro-4*H*-pyrido[1,2-*a*]pyrimidin-4-one (14)

White amorphous solid, m. p. = 91.0 °C. IR (thin film, cm^−1^): 1624, 1483, 1366. ^1^H NMR (300 MHz, DMSO-d_6_): δ 8.07 (d, J = 7.0 Hz, 2 H), 6.74 (t, J = 1.0 Hz, 1 H), 4.38 (t, J = 7.3 Hz, 2 H), 2.54–2.49 (m, 2 H); ^13^C NMR (75 MHz, DMSO-d_6_): δ = 174.1, 154.0, 144.2, 140.1, 140.0, 118.8, 109.2, 50.0, 29.0. ESI Mass (m/z): calcd for C_9_H_8_F_3_N_2_O [M + H]^+^ 217.0, found 217.0.

### 9-Bromo-7-methyl-2,3-dihydro-4*H*-pyrido[1,2-*a*]pyrimidin-4-one (15)

Brownish solid, m. p. = 115.2 °C. IR (thin film, cm^−1^): 1664, 1587, 1478, 603. ^1^H NMR (300 MHz, DMSO-d_6_): δ 8.01 (d, J = 1.6 Hz, 1 H), 7.76 (s, 1 H), 4.29 (t, J = 7.3 Hz, 2 H), 2.44 (t, J = 7.2 Hz, 2 H), 2.11 (s, 3 H); ^13^C NMR (75 MHz, DMSO-d_6_): δ = 174.6, 153.5, 145.3, 137.6, 120.6, 115.7, 50.7, 29.5, 16.6. HRMS (ESI-FTMS Mass (m/z): calcd for C_9_H_10_BrN_2_O [M + H]^+^ 242.9898, found 242.9889.

### 7-Bromo-9-methyl-2,3-dihydro-4*H*-pyrido[1,2-*a*]pyrimidin-4-one (16)

Yellowish amorphous solid, m. p. = 110.8 °C. IR (thin film, cm^−1^): 1640, 1604, 1583, 1482, 603. ^1^H NMR (300 MHz, CDCl_3_): δ 7.45 (d, J = 1.1 Hz, 1 H), 7.37 (s, 1 H), 4.26 (t, J = 7.2 Hz, 2 H), 2.73 (t, J = 7.3 Hz, 2 H), 2.31 (s, 3 H); ^13^C NMR (75 MHz, CDCl_3_): δ = 173.6, 157.8, 147.6, 160.5, 159.3, 157.8, 157.2, 50.7, 19.7, 19.0.; HRMS (ESI-FTMS Mass (m/z): calcd for C_9_H_10_BrN_2_O [M + H]^+^ 242.9898, found 242.9913.

### 7-Bromo-8-methyl-2,3-dihydro-4*H*-pyrido[1,2-*a*]pyrimidin-4-one (17)

White solid, m. p. = 130.5 °C. IR (thin film, cm^−1^): 1641, 1593, 530. ^1^H NMR (300 MHz, CDCl_3_): δ 7.71 (s, 1 H), 6.76 (s, 1 H), 4.31 (t, J = 7.2 Hz, 2 H), 2.64 (t, J = 7.4 Hz, 2 H), 2.30 (s, 3 H); ^13^C NMR (75 MHz, CDCl_3_): δ = 169.8, 151.7, 147.7, 133.0, 117.4, 104.1, 45.3, 24.3, 17.9. HRMS (ESI-FTMS Mass (m/z): calcd for C_9_H_10_BrN_2_O [M + H]^+^ 242.9898, found 242.9899.

### 7-Iodo-9-methyl-2,3-dihydro-4*H*-pyrido[1,2-*a*]pyrimidin-4-one (18)

Brown solid, m. p. = 138.3 °C. IR (thin film, cm^−1^): 1640, 1483, 537. ^1^H NMR (300 MHz, DMSO-d_6_): δ 8.52 (s, 1 H), 8.22 (s, 1 H), 4.53 (br s, 2 H), 2.72 (br s, 2 H), 2.20 (s, 3 H); ^13^C NMR (75 MHz, DMSO-d_6_): δ = 170.7, 150.9, 150.3, 149.6, 143.2, 129.2, 50.4, 29.1, 17.1. ESI Mass (m/z): calcd for C_9_H_9_IN_2_O [M + H]^+^ 288.9, found 288.9.

### Methyl 3-[(6-methylpyridin-2-yl)amino]propanoate (19)

Viscous liquid, ^1^H NMR (300 MHz, CDCl_3_): δ 7.34–7.27 (m, 1 H), 6.45 (d, J = 7.2 Hz, 1 H), 6.21 (d, J = 8.2 Hz, 1 H), 3.69 (s, 1 H), 3.60 (q, J = 6.3 Hz, 2 H), 2.64 (t, J = 6.3 Hz, 2 H), 2.38 (s, 3 H); ^13^C NMR (75 MHz, CDCl_3_): δ = 172.8, 157.8, 157.0, 137.7, 112.3, 103.4, 51.7, 37.6, 34.0, 24.3. ESI Mass (m/z): calcd for C_10_H_15_N_2_O_2_ [M + H]^+^ 165.1, found 165.1.

### 3-[(4-chloropyridin-2-yl)amino]propanoic acid (20)

White solid powder, m. p. = 283.3 °C. IR (thin film, cm^−1^): 3360, 1710, 1483. ^1^H NMR (300 MHz, DMSO-d_6_): δ 8.78 (d, J = 7.3 Hz, 1 H), 7.68 (d, J = 4.2 Hz, 1 H), 7.23 (s, 1 H), 4.71 (t, J = 6.9 Hz, 2 H), 2.96 (t, J = 7.0 Hz, 2 H); ^13^C NMR (75 MHz, DMSO-d_6_): δ = 171.8, 171.6, 126.0, 150.3, 112.0, 105.6, 54.7, 33.7. ESI Mass (m/z): calcd for C_8_H_7_ClN_2_O [M + H]^+^ 183.0, found 184.0.

## Additional Information

**How to cite this article**: Alam, M. A. *et al*. Hexafluoroisopropyl alcohol mediated synthesis of 2,3-dihydro-4H-pyrido[1,2-a]pyrimidin-4-ones. *Sci. Rep*. **6**, 36316; doi: 10.1038/srep36316 (2016).

**Publisher’s note:** Springer Nature remains neutral with regard to jurisdictional claims in published maps and institutional affiliations.

## Supplementary Material

Supplementary Information

## Figures and Tables

**Figure 1:**
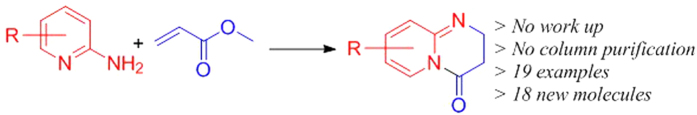
Synthesis of dihydropyrido-pyrimidinones.

**Figure 2:**
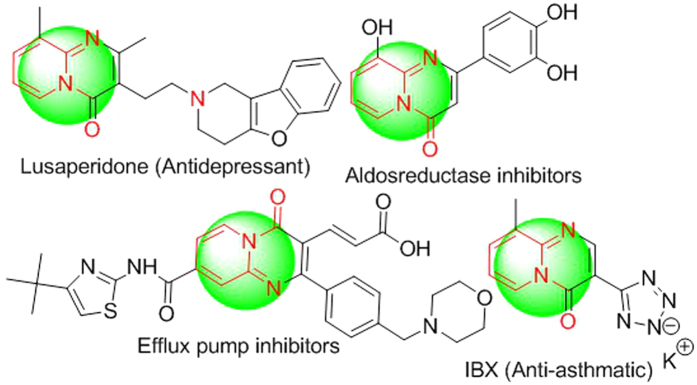
Pyrido-pyrimidones bearing marketed drug and drug targets.

**Figure 3:**
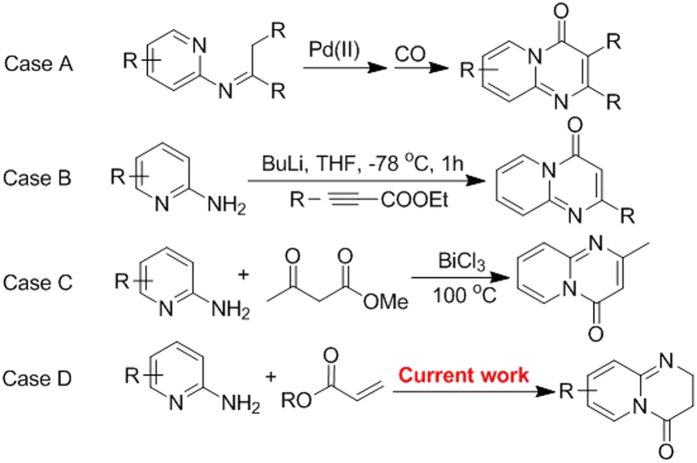
Strategies for the synthesis of pyrido-pyrimidinone and their derivatives.
