# Supplementary material for: Hexafluoroisopropyl alcohol mediated synthesis of 2,3-dihydro-4H-pyrido[1,2-a]pyrimidin-4-ones
Source: Sci Rep. 2016 Nov 2;6:36316. doi: 10.1038/srep36316 (PMC5090868; doi:10.1038/srep36316)

Hexafluoroisopropyl alcohol mediated synthesis of 2,3-dihydro-4H-pyrido[1,2-a]pyrimidin-4-ones

Mohammad A. Alam,* Zakeyah Alsharif[a], Hessa Alkhattabi[a], Derika Jones[a], Evan Delancey[a], Adam Gottsponer[a], Tianhong Yang[b]

[a] Department of Chemistry and Physics, College of Science and Mathematics, Arkansas State University, Jonesboro, AR 72467
[b]Molecular Biosciences Graduate Program, Arkansas State University, Jonesboro, AR 72467
*corresponding author

Mohammad A Alam

E-mail: malam@astate.edu, Tel: +1-870-973-3319

Materials and instrumentation

All commercial chemicals and solvents are reagent grade and were used without further treatment unless otherwise noted. ^{1}H NMR spectra were obtained with a Varian Mercury-300MHz with TMS as internal standard. ^{13}C NMR spectra were obtained with a Varian Mercury-75MHz with TMS as internal standard. 13C NMR were obtained by proton-decoupled data. IR spectra were recorded in Thermo Scientific NICOLET IS10. The ESI-FTMS Mass spectra were recorded Bruker ApexII-FTMS system. Low resolution mass spectra were recorded in Shimadzu QQQ instrument and LTQ-XL mass spectrometer. Melting points were recorded in MP 50 melting point system.

^{1}H NMR spectrum of **2**

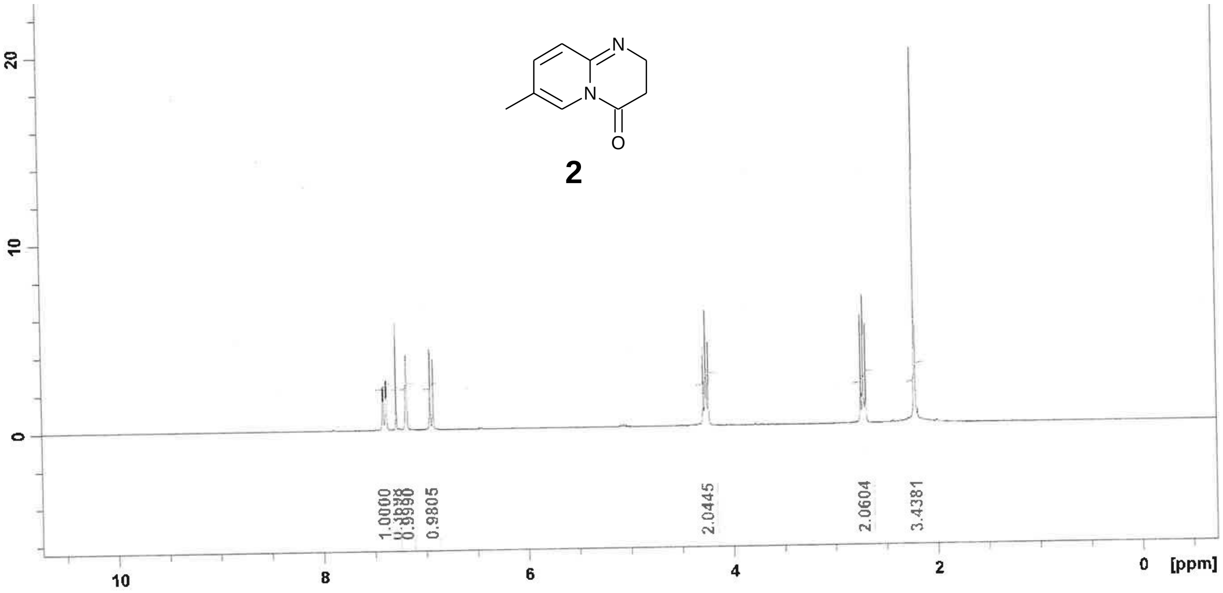

^{13}C NMR spectrum of **2**

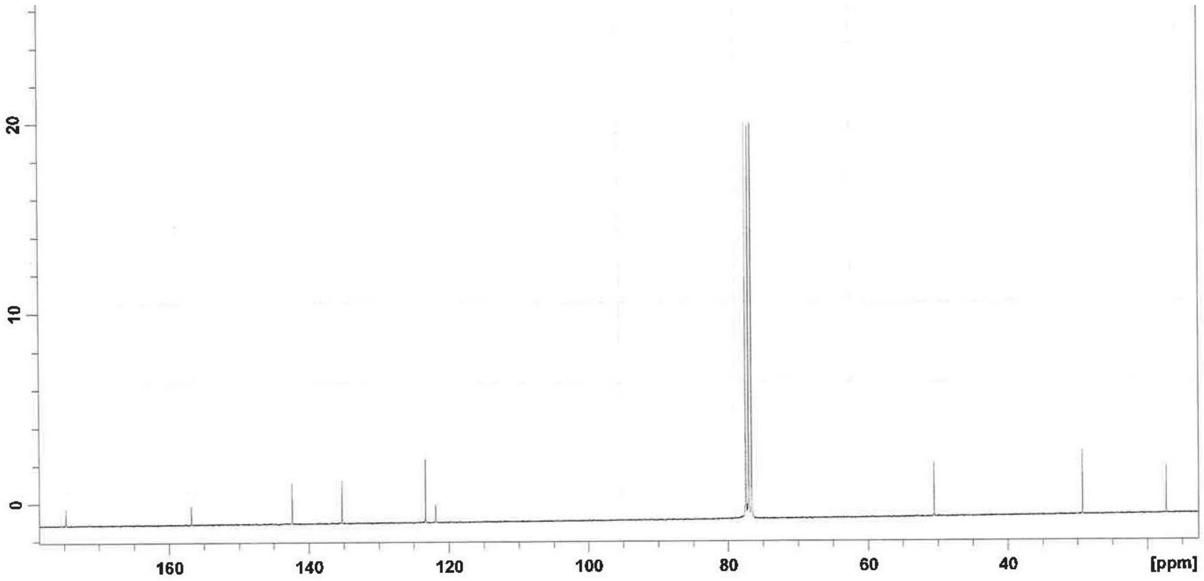

^{1}H NMR spectrum of **3**

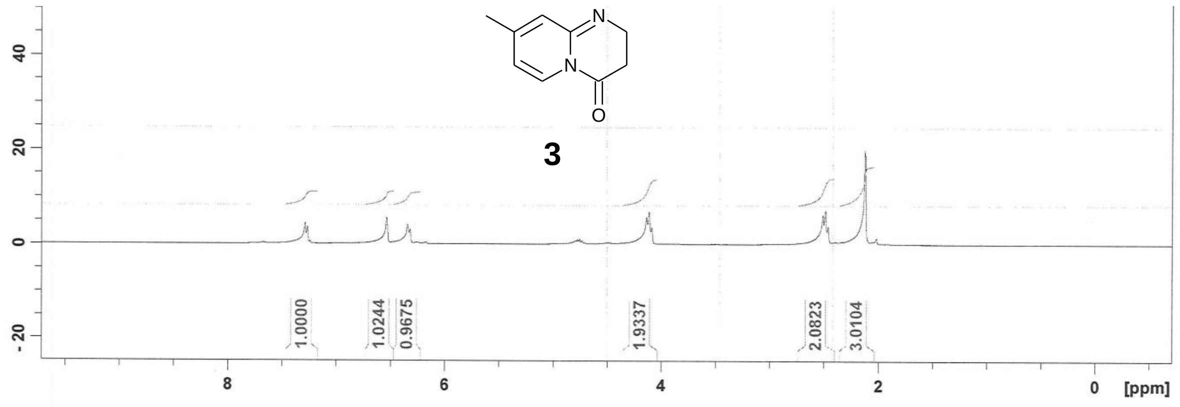

^{13}C NMR spectrum of **3**

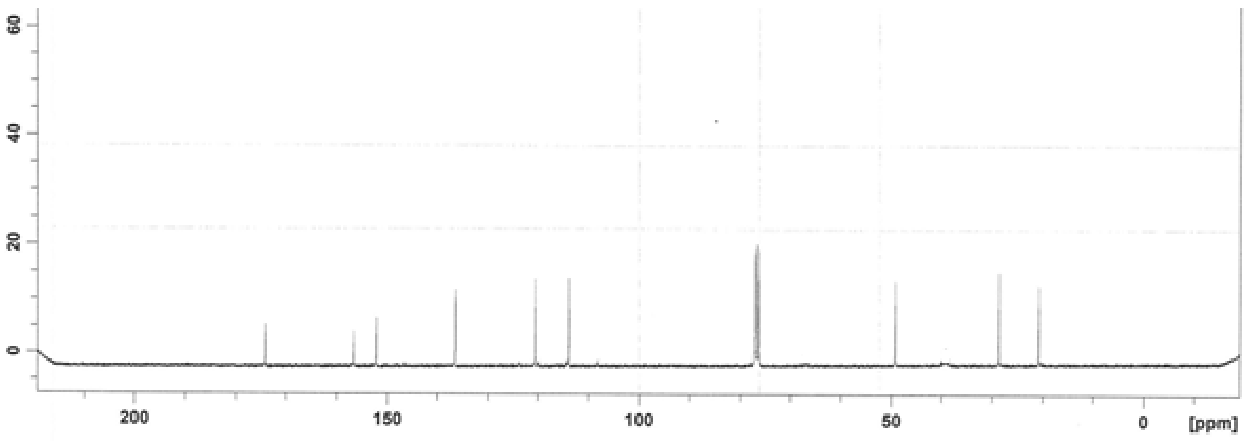

^{1}H NMR spectrum of **4**

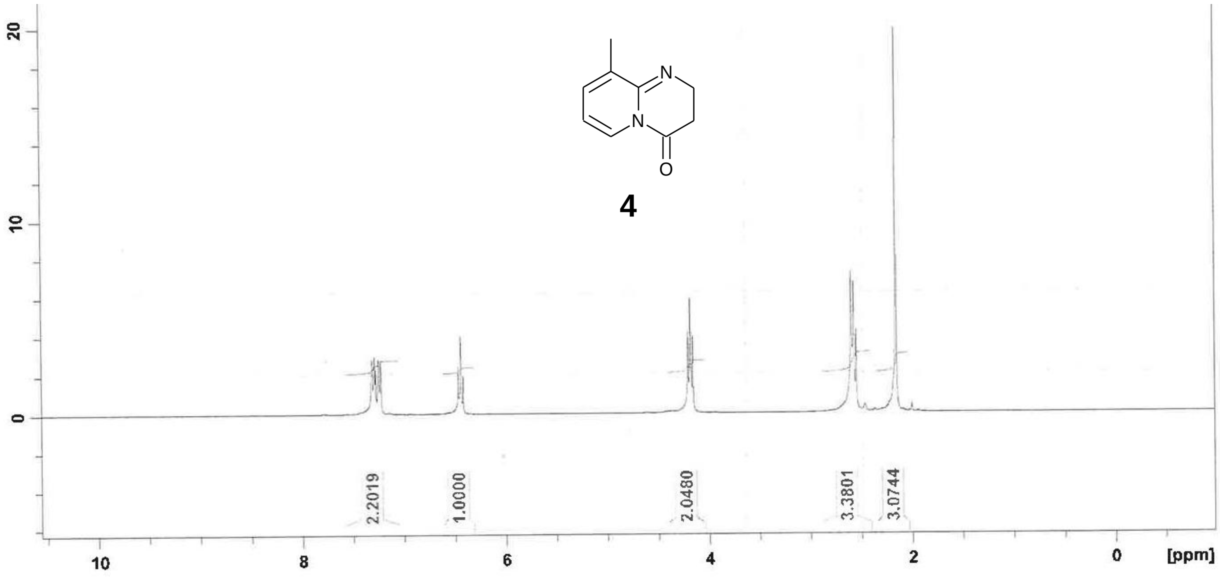

^{13}C NMR spectrum of **4**

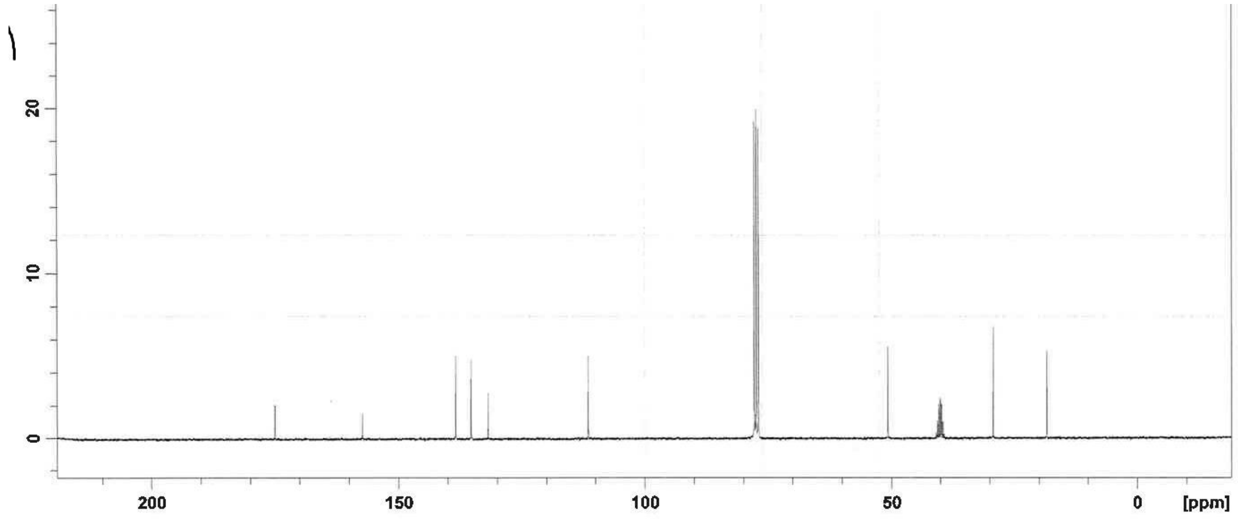

^{1}H NMR spectrum of **5**

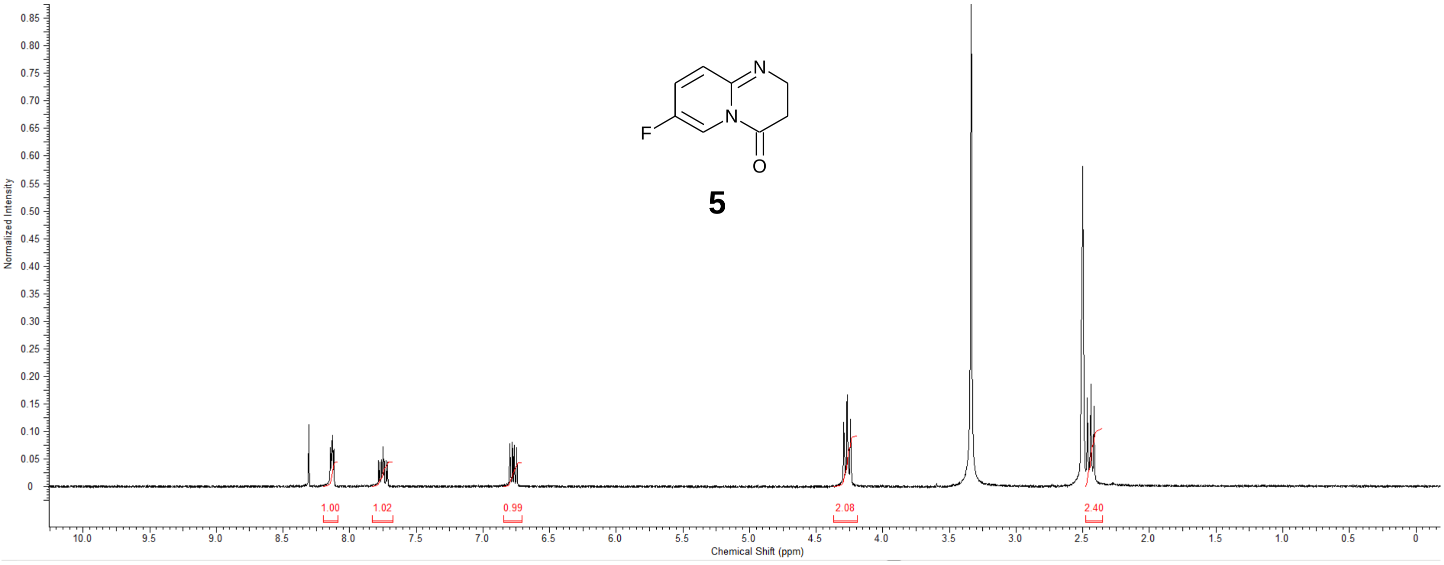

^{13}C NMR spectrum of **5**

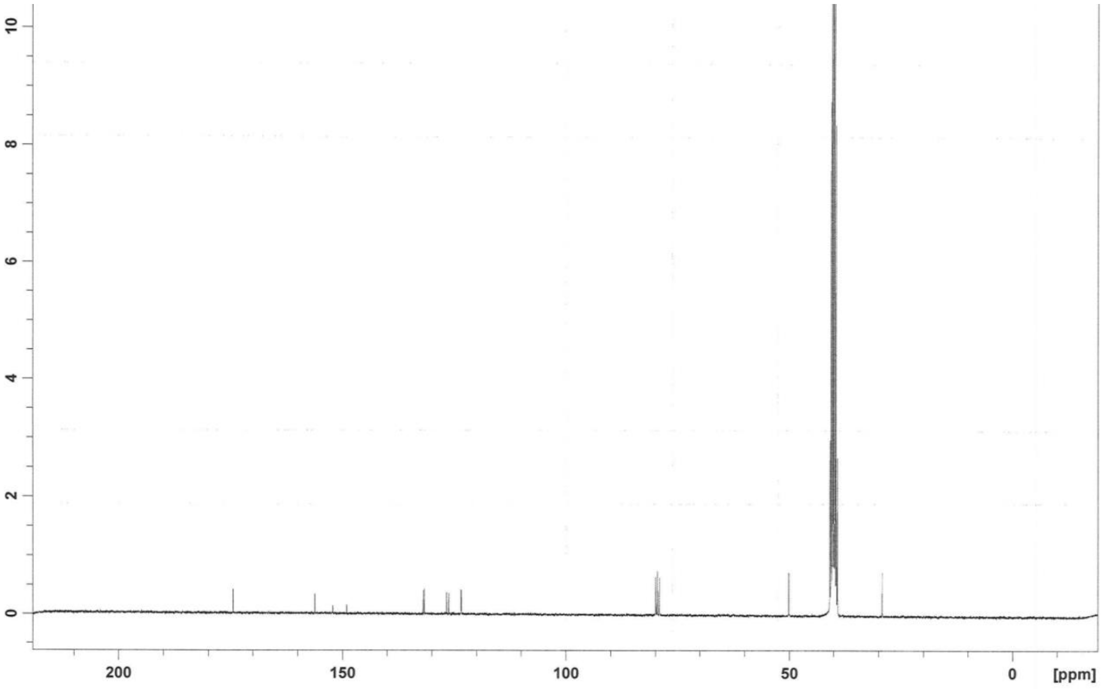

^{1}H NMR spectrum of **6**

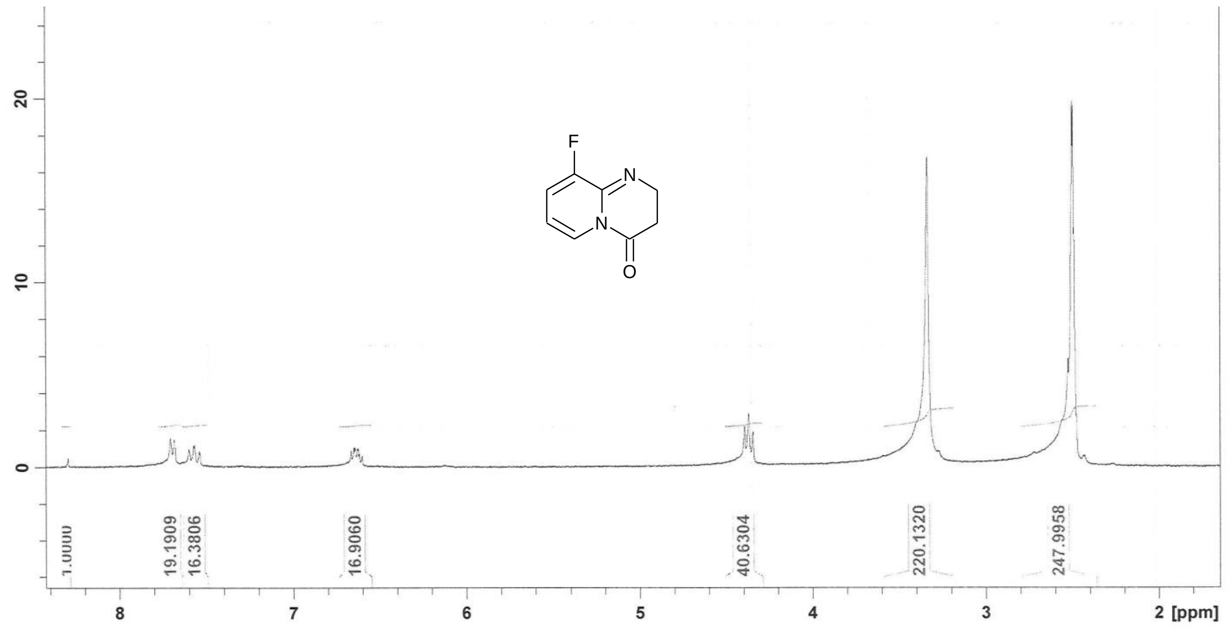

^{13}C NMR spectrum of **6**

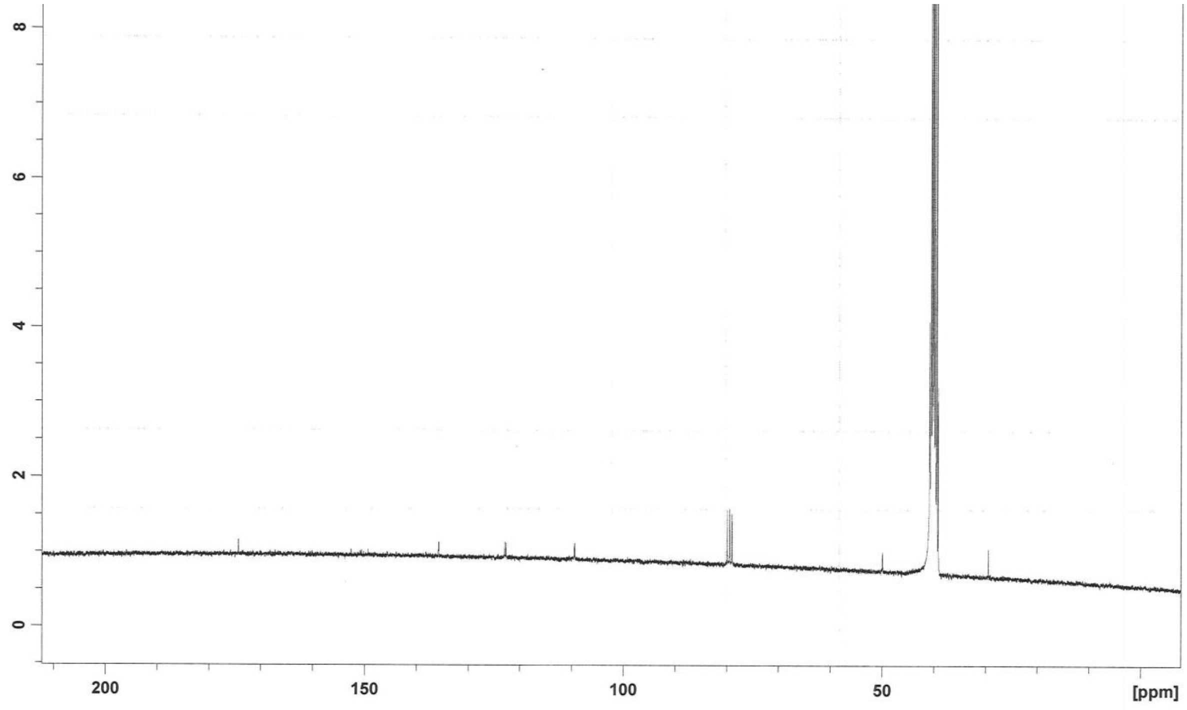

^{1}H NMR spectrum of 7

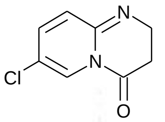

7

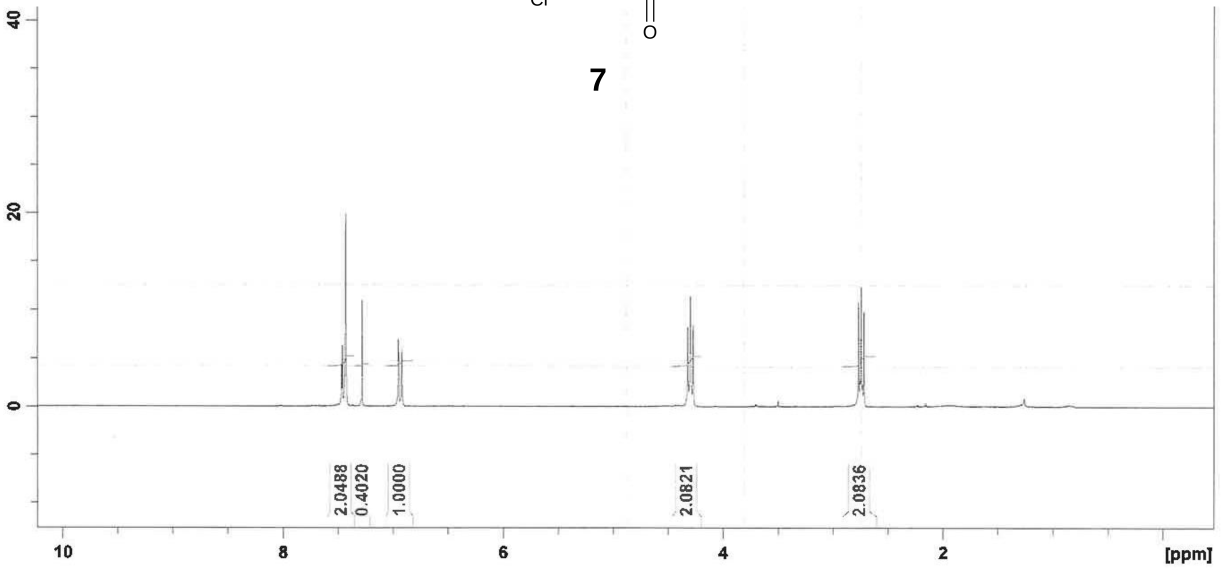

^{13}C NMR spectrum of **7**

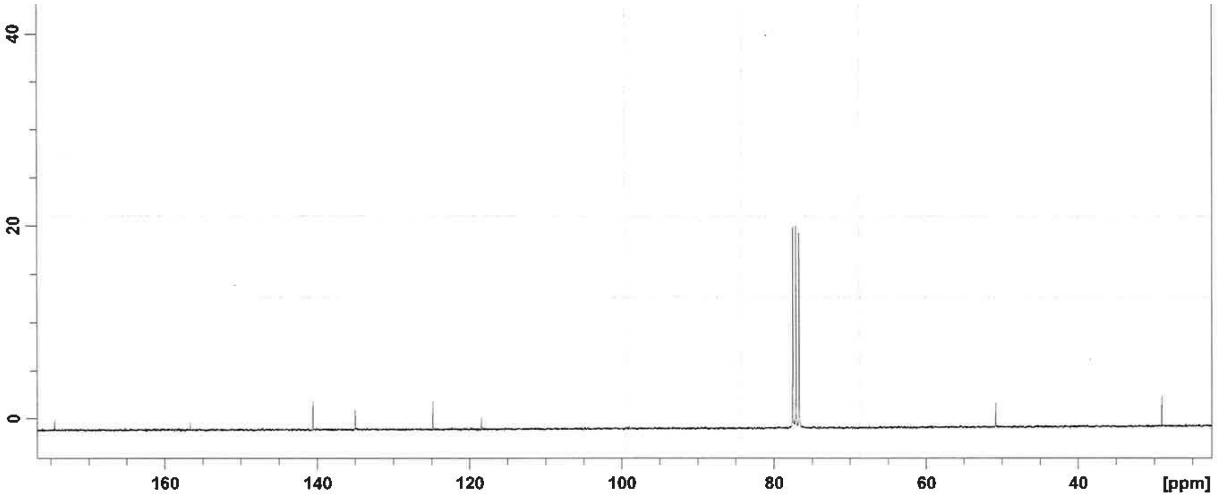

^{1}H NMR spectrum of **8**

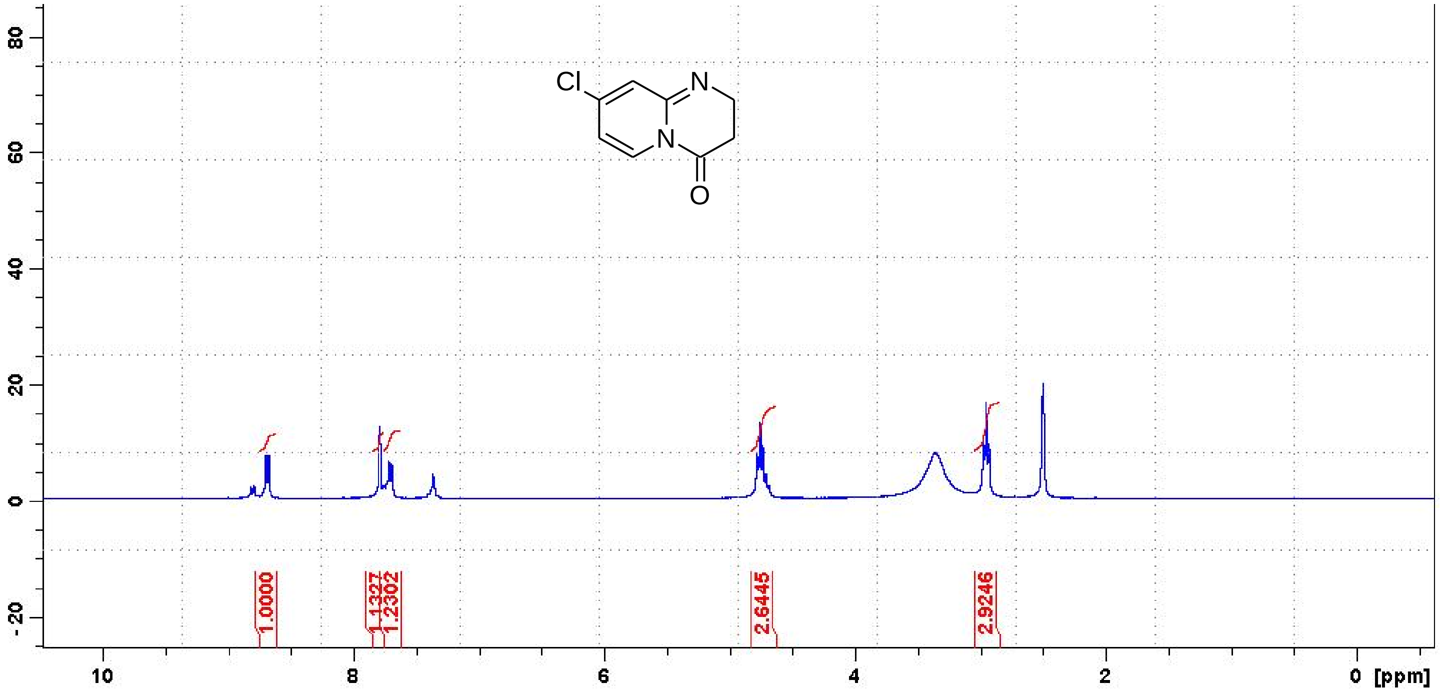

^{13}C NMR spectrum of **8**

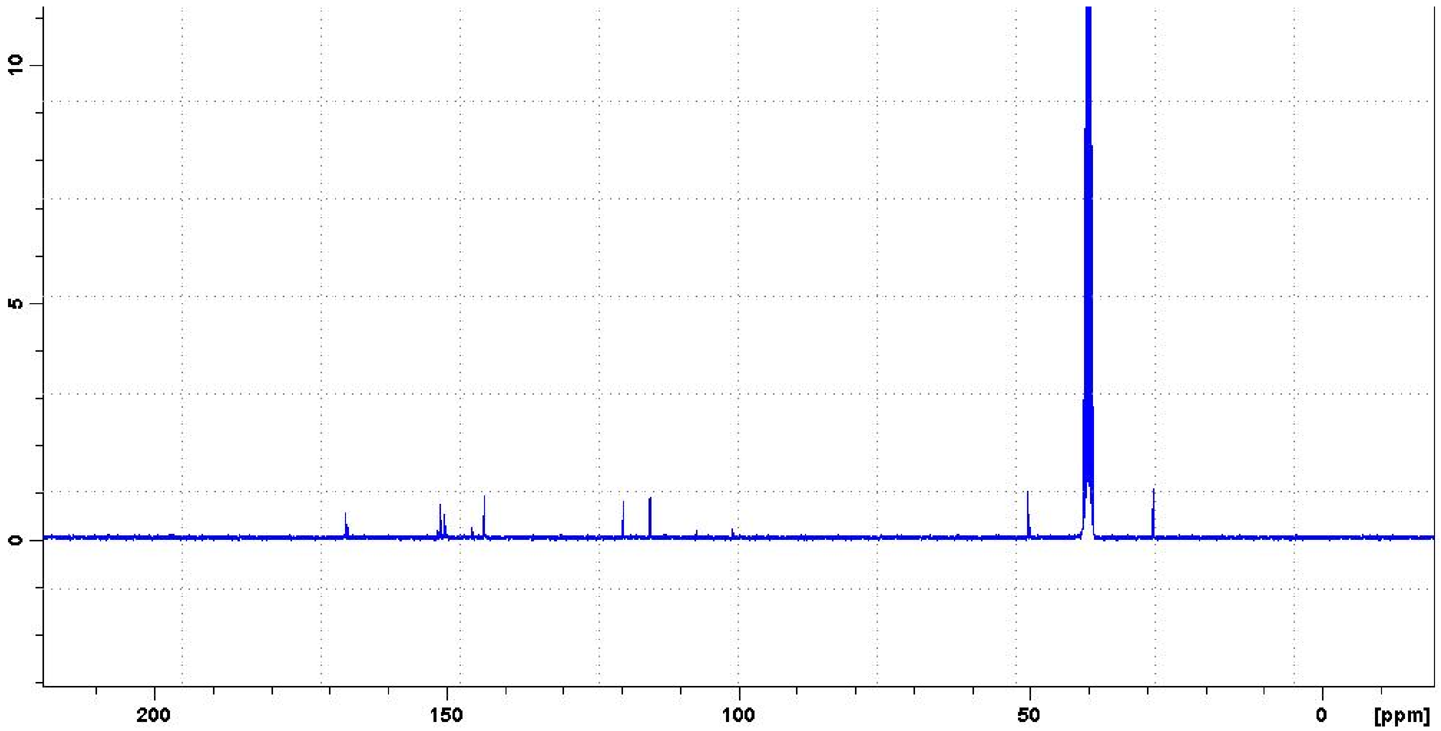

^{1}H NMR spectrum of **9**

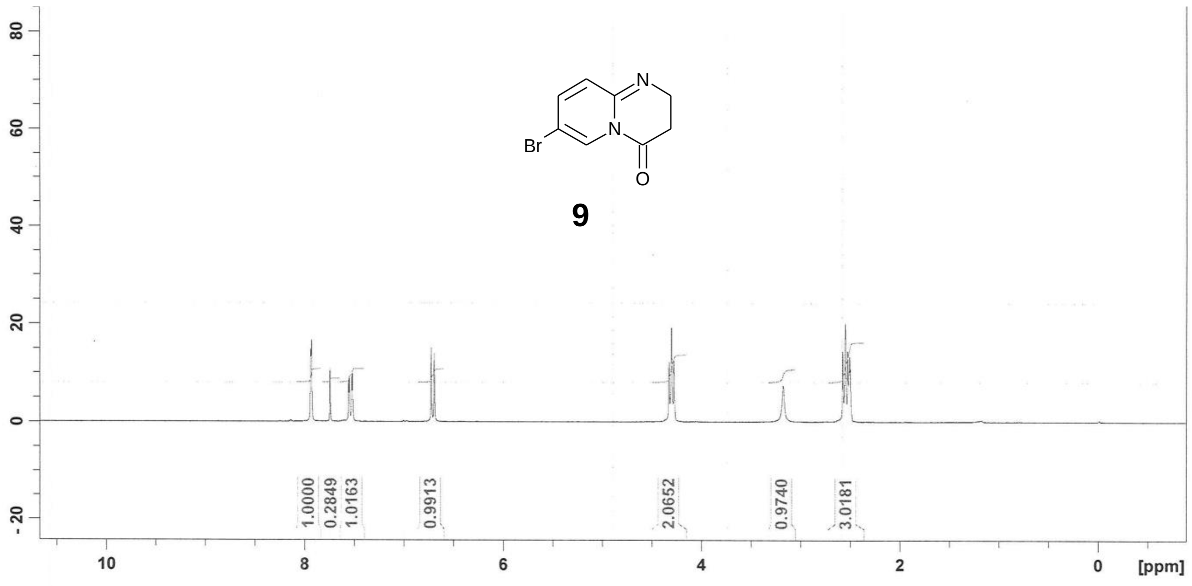

^{13}C NMR spectrum of **9**

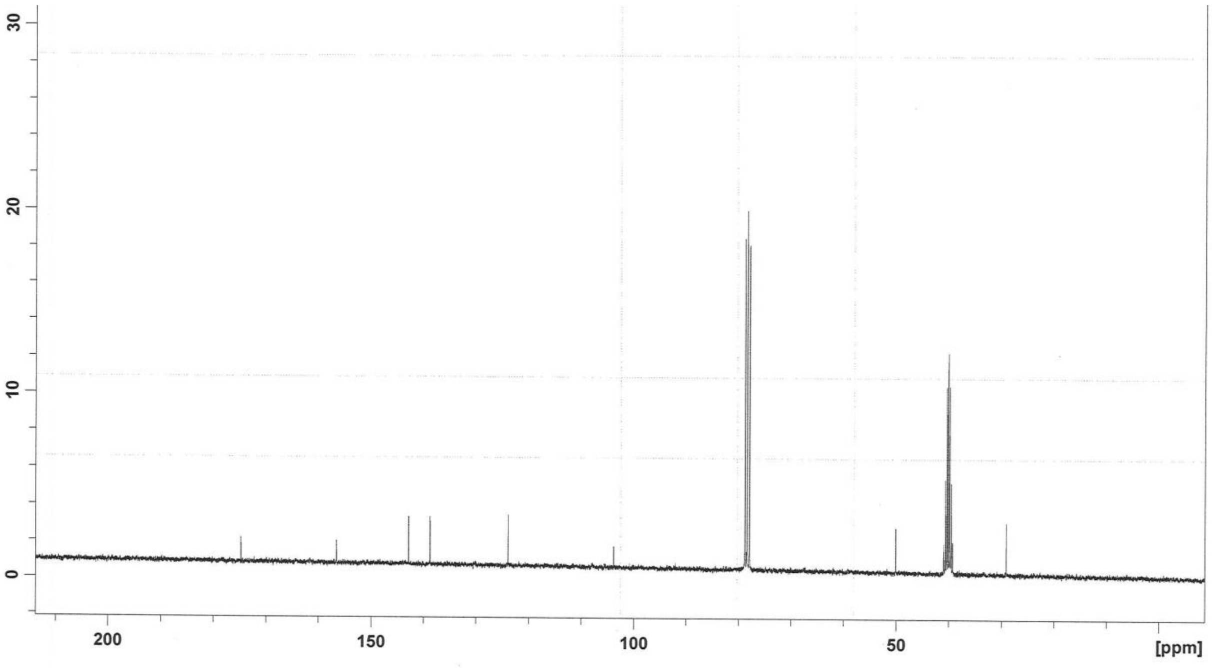

^{1}H NMR spectrum of **10**

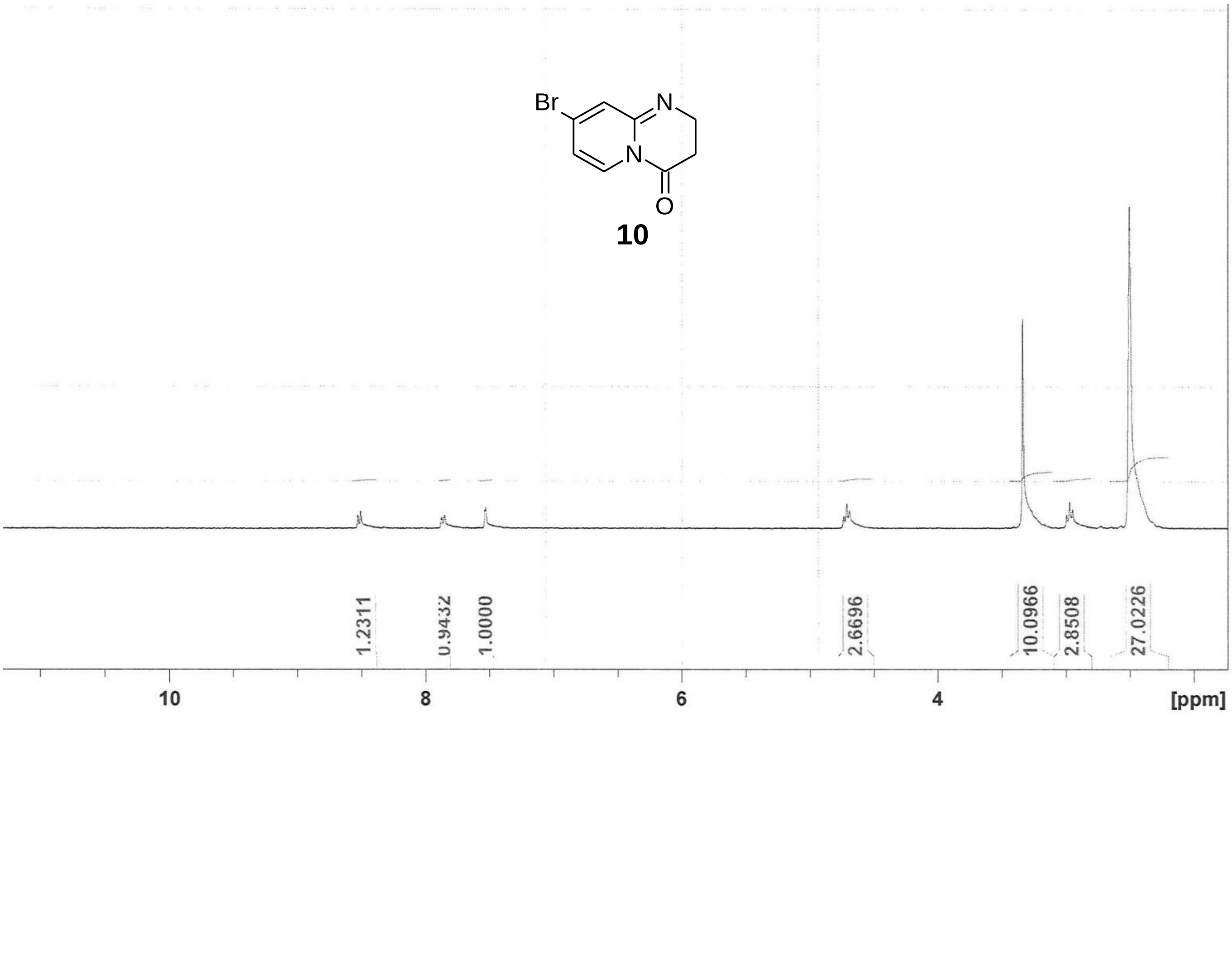

^{13}C NMR spectrum of **10**

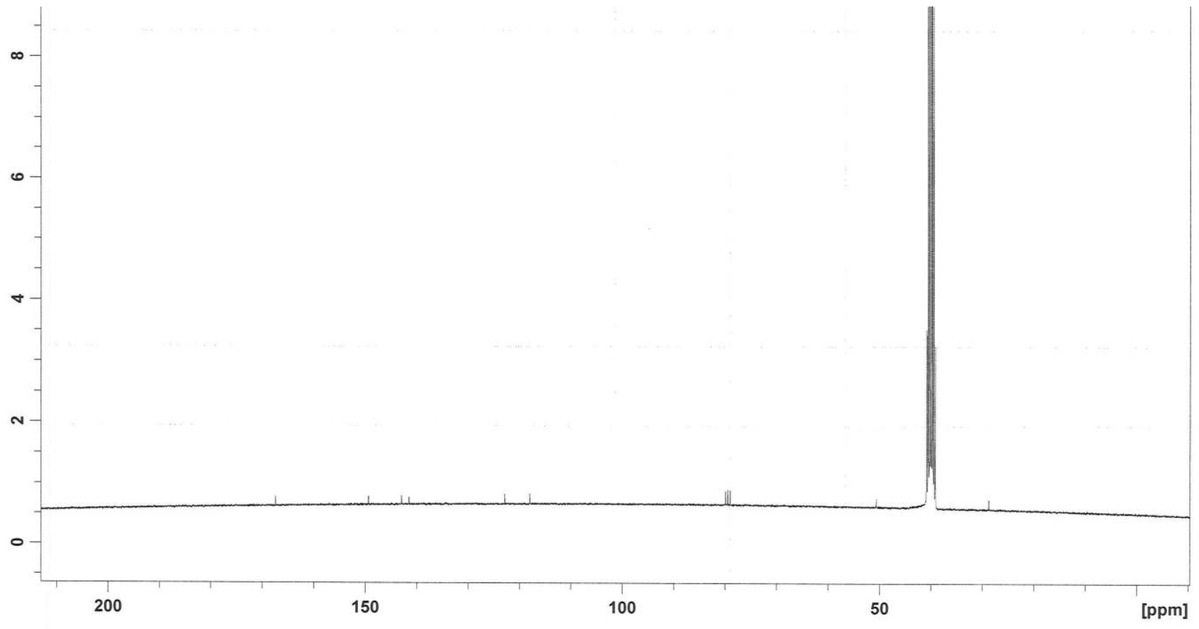

^{1}H NMR spectrum of **11**

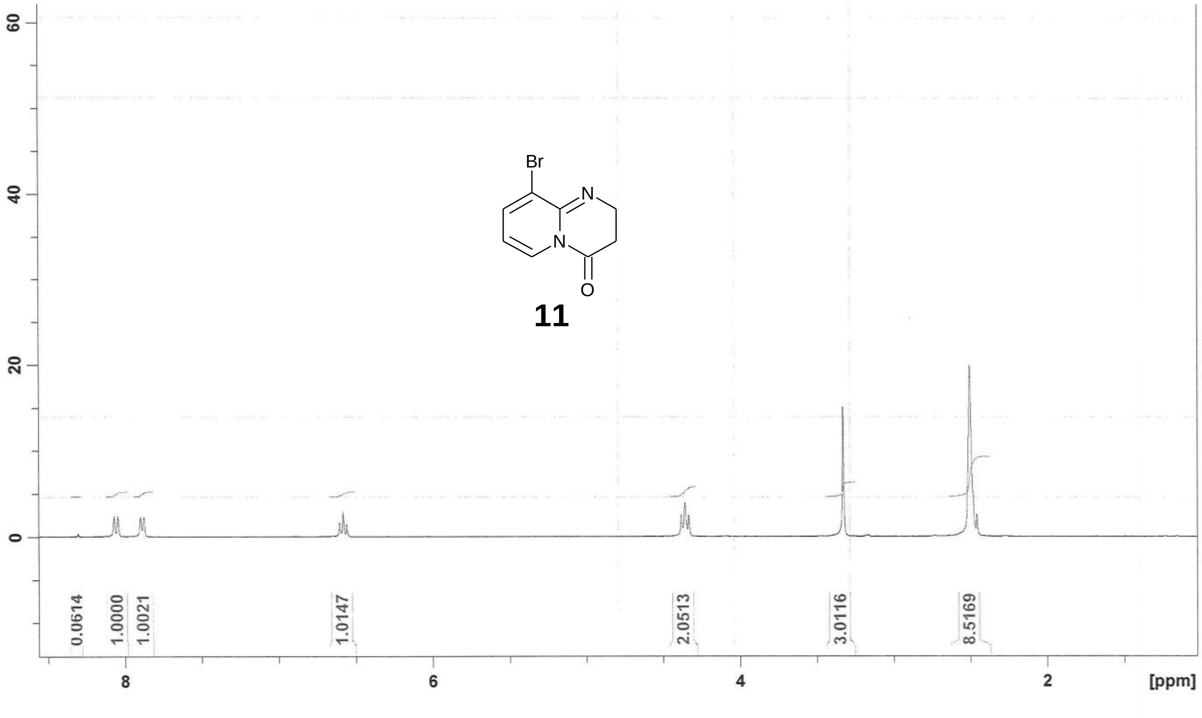

^{13}C NMR spectrum of **11**

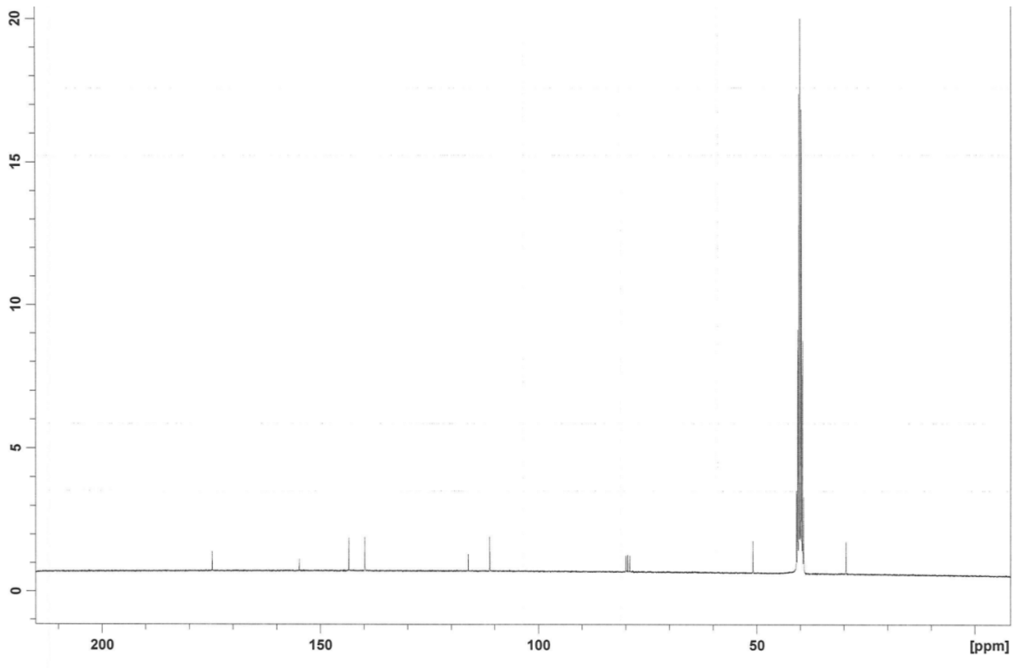

.

^{1}H NMR spectrum of **12**

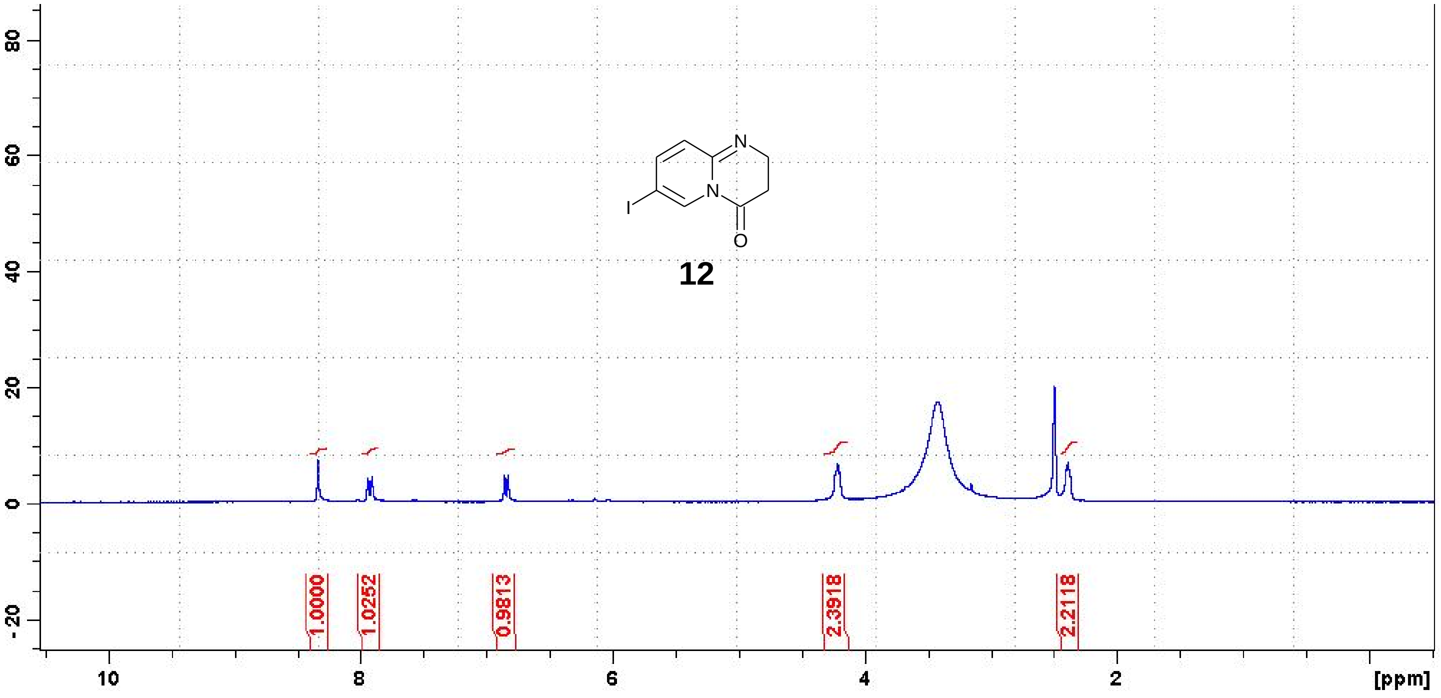

^{13}C NMR spectrum of **12**

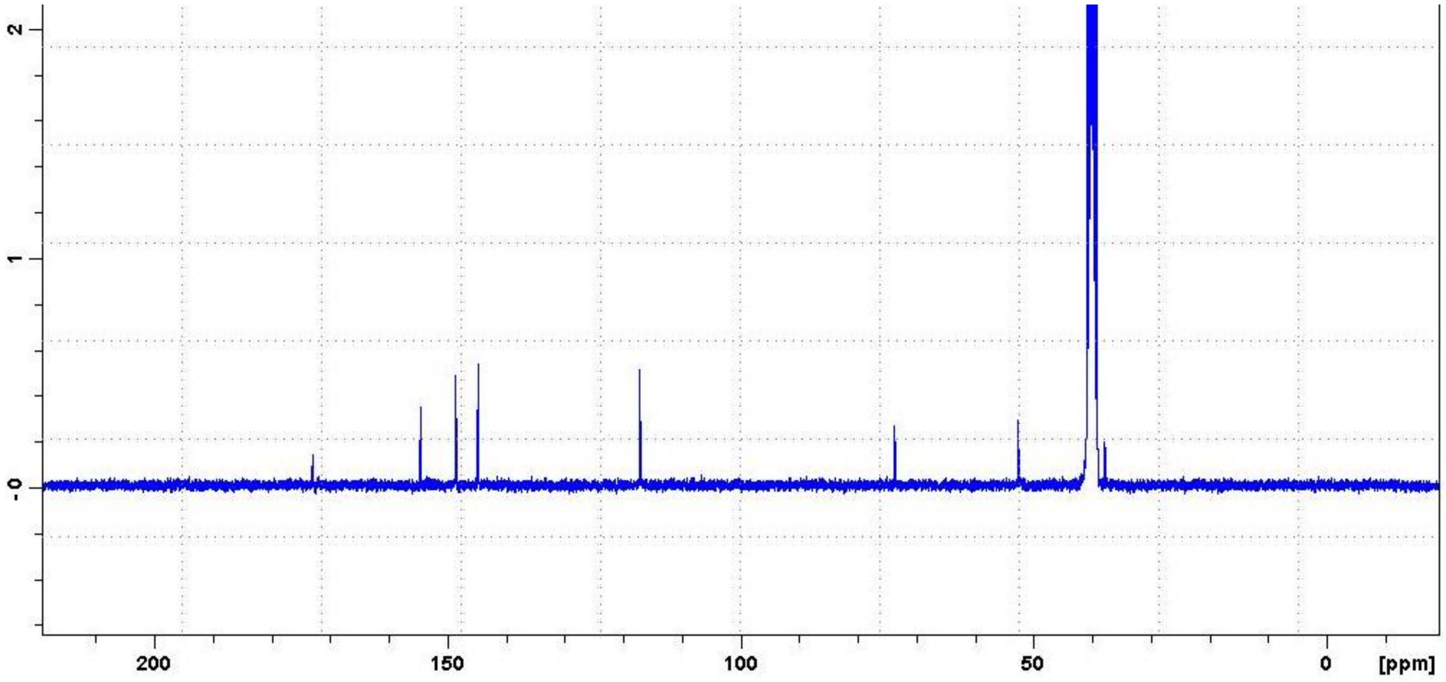

^{1}H NMR spectrum of **13**

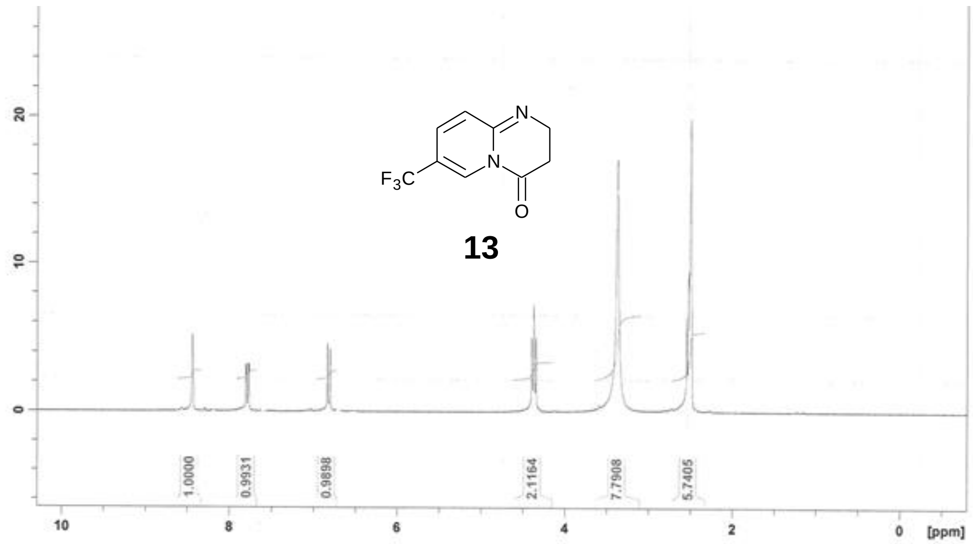

^{13}C NMR spectrum of **13**

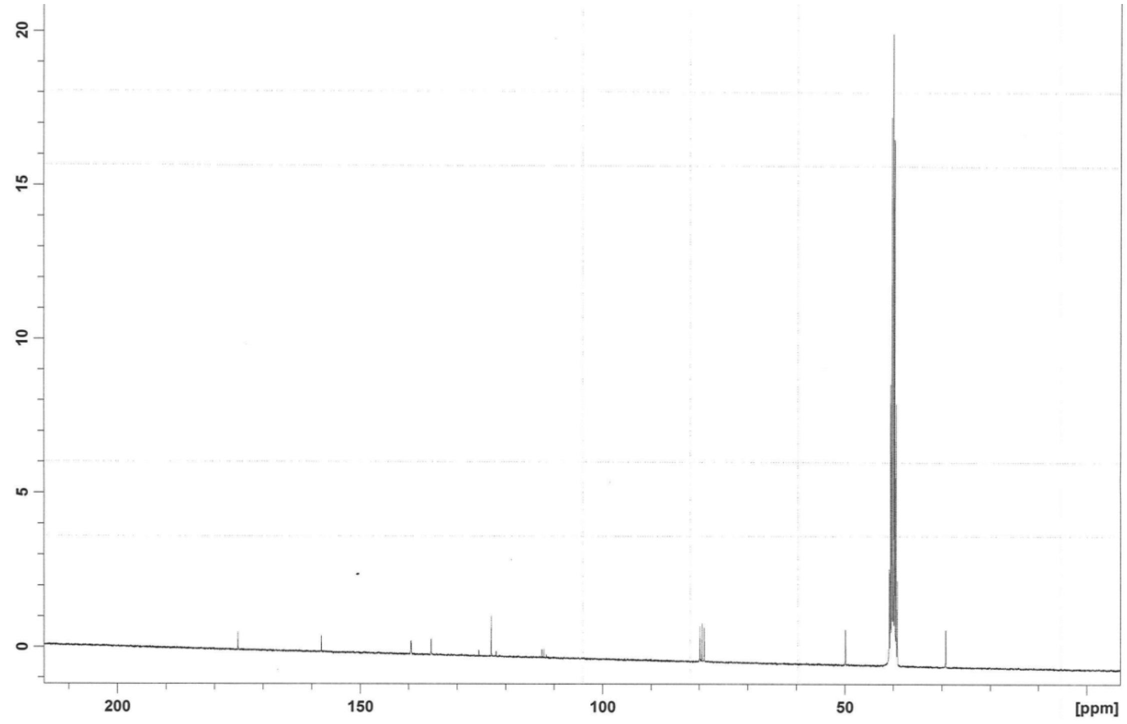

^{1}H NMR spectrum of **14**

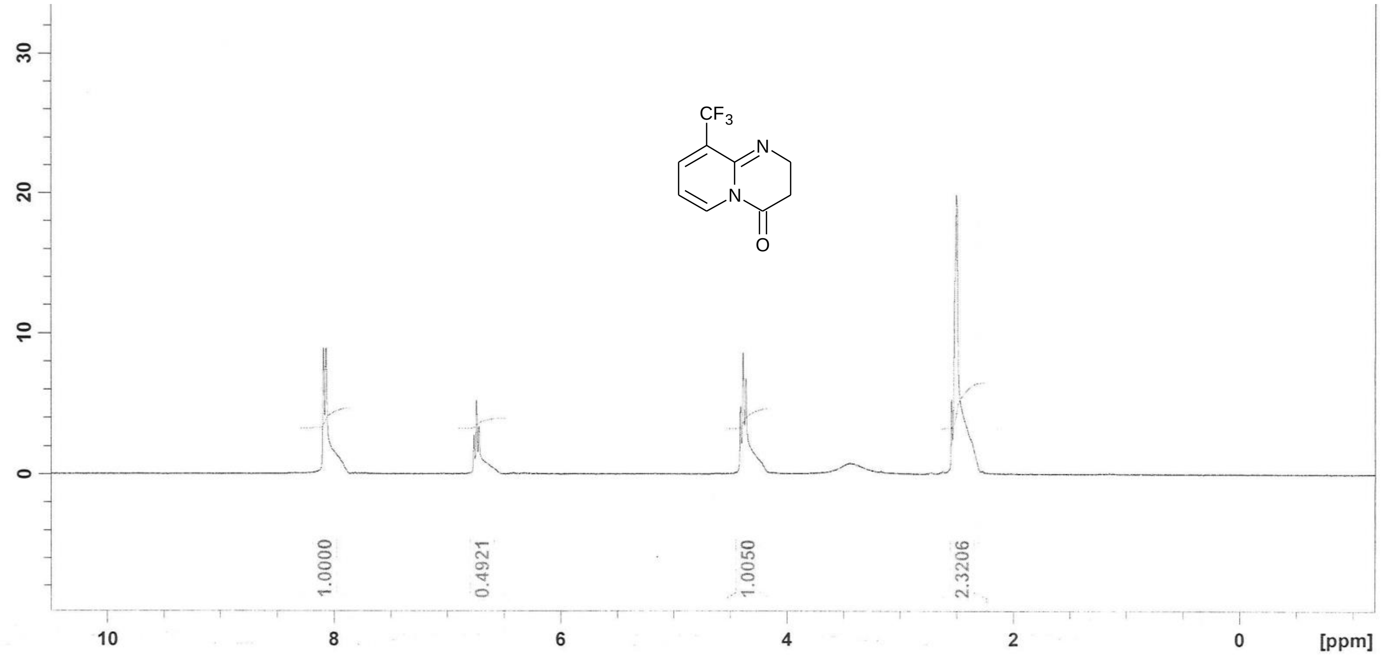

^{13}C NMR spectrum of **14**

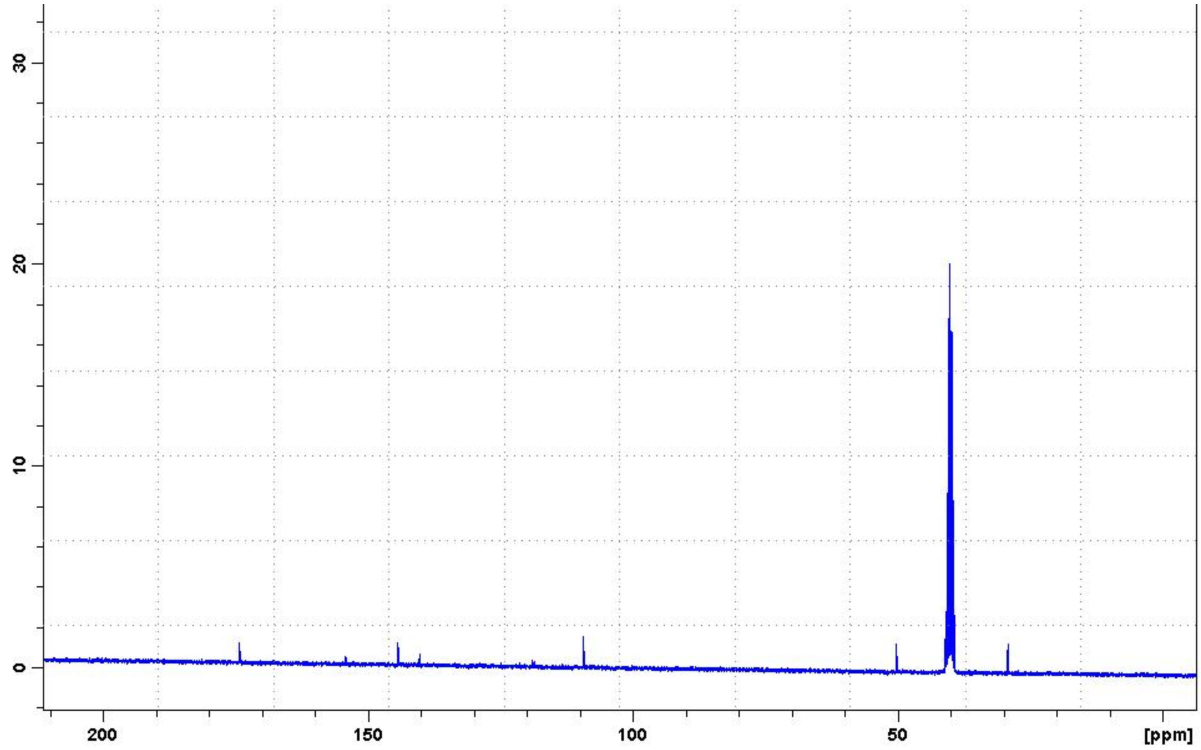

^{1}H NMR spectrum of **15**

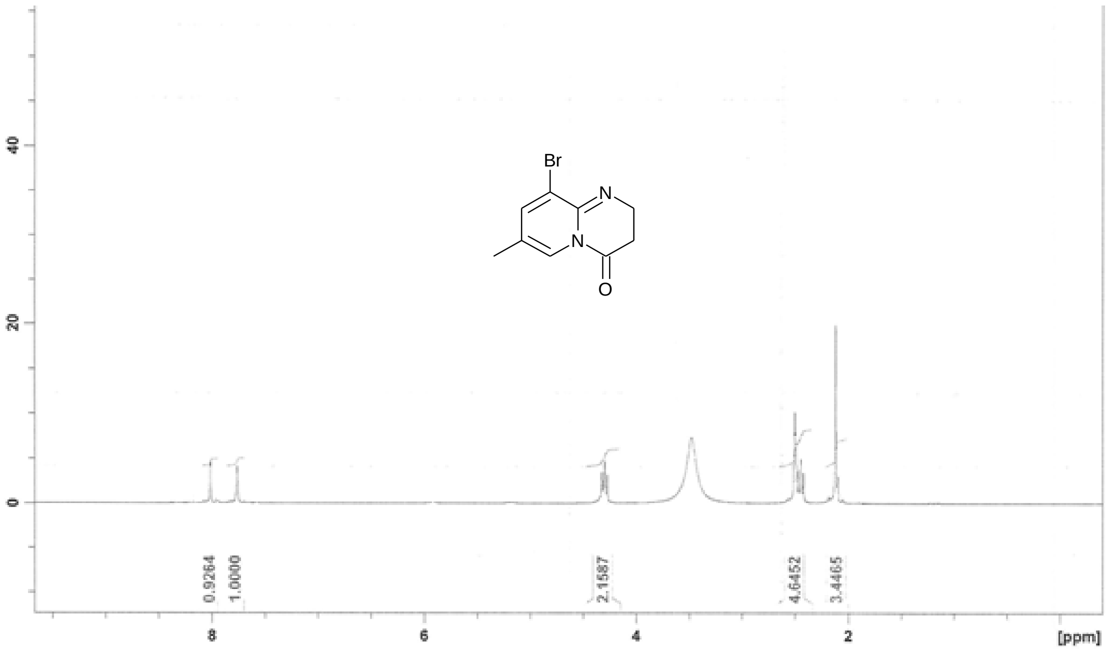

^{13}C NMR spectrum of **15**

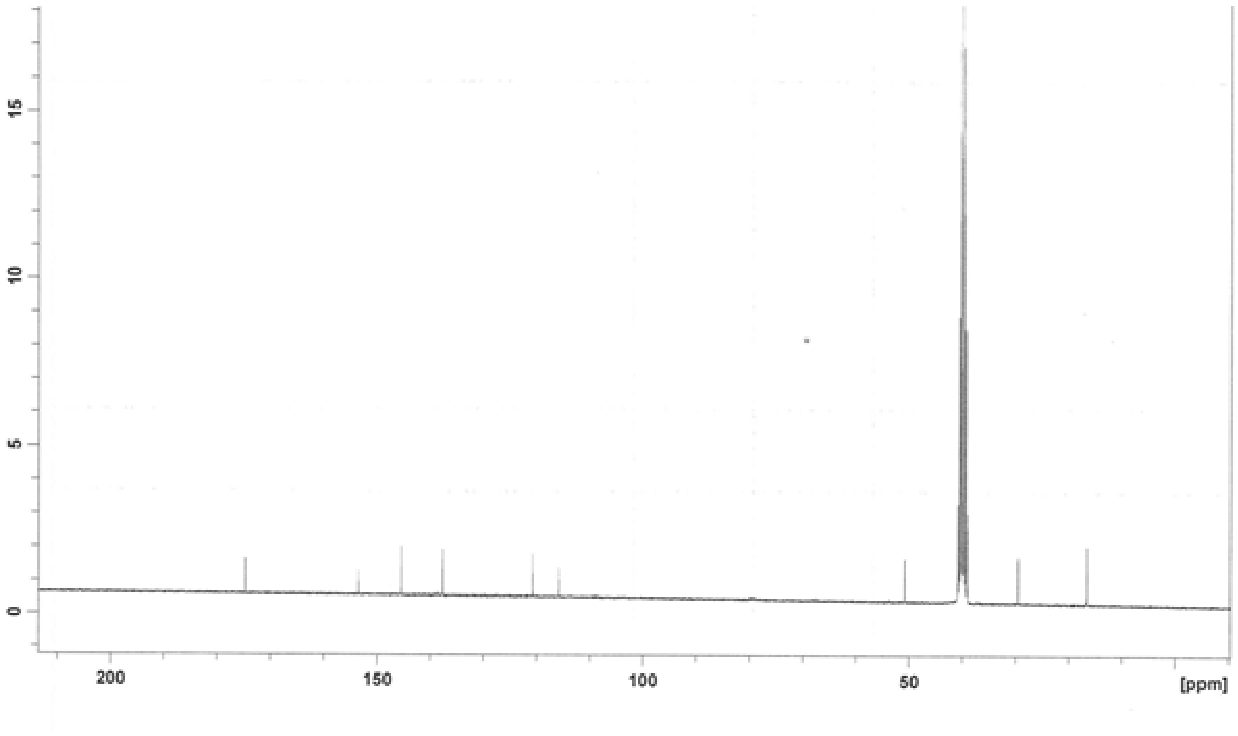

^{1}H NMR spectrum of **16**

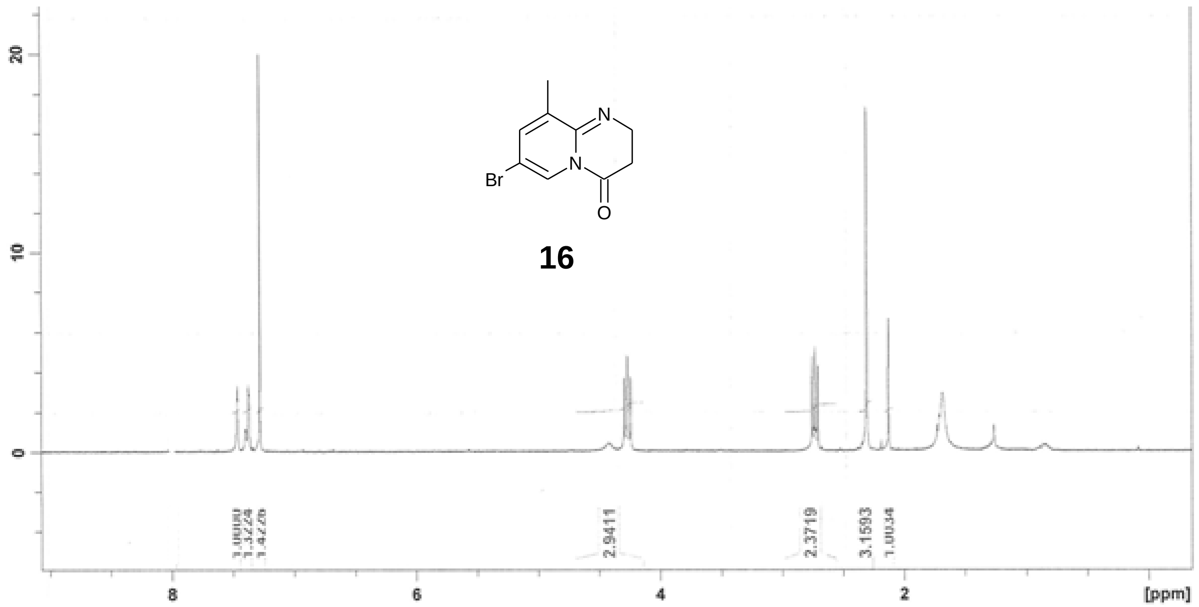

^{13}C NMR spectrum of **16**

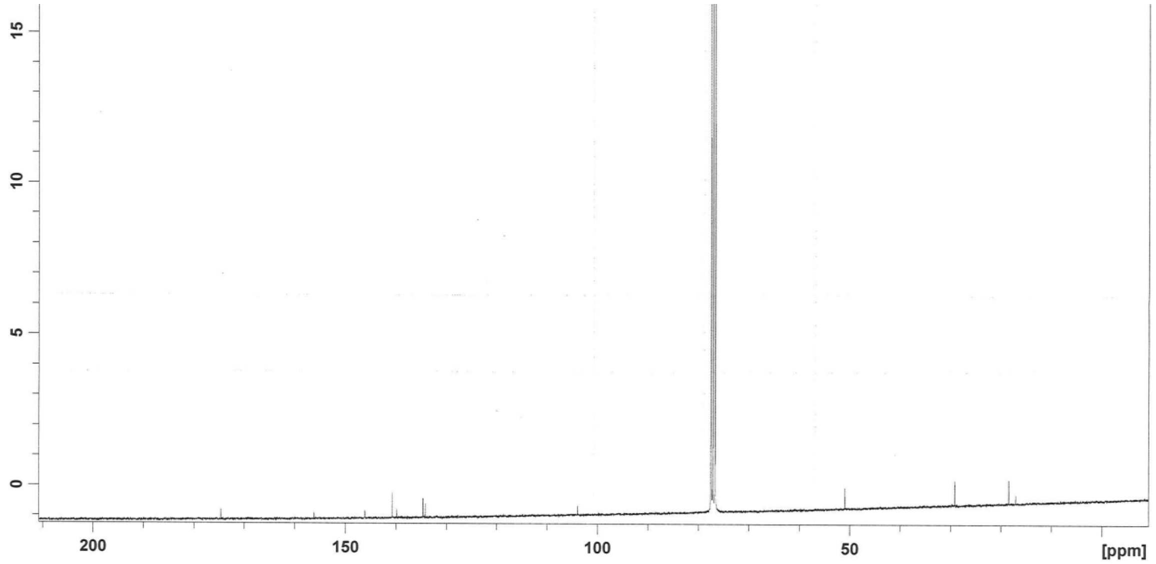

^{1}H NMR spectrum of **17**

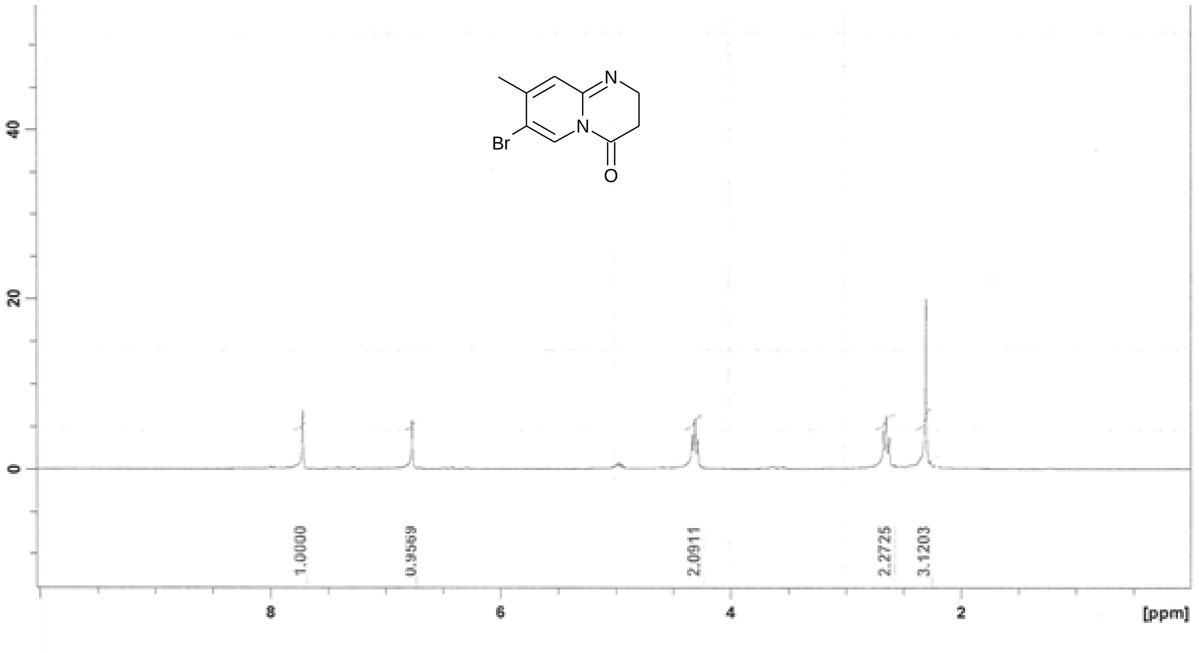

^{13}C NMR spectrum of **17**

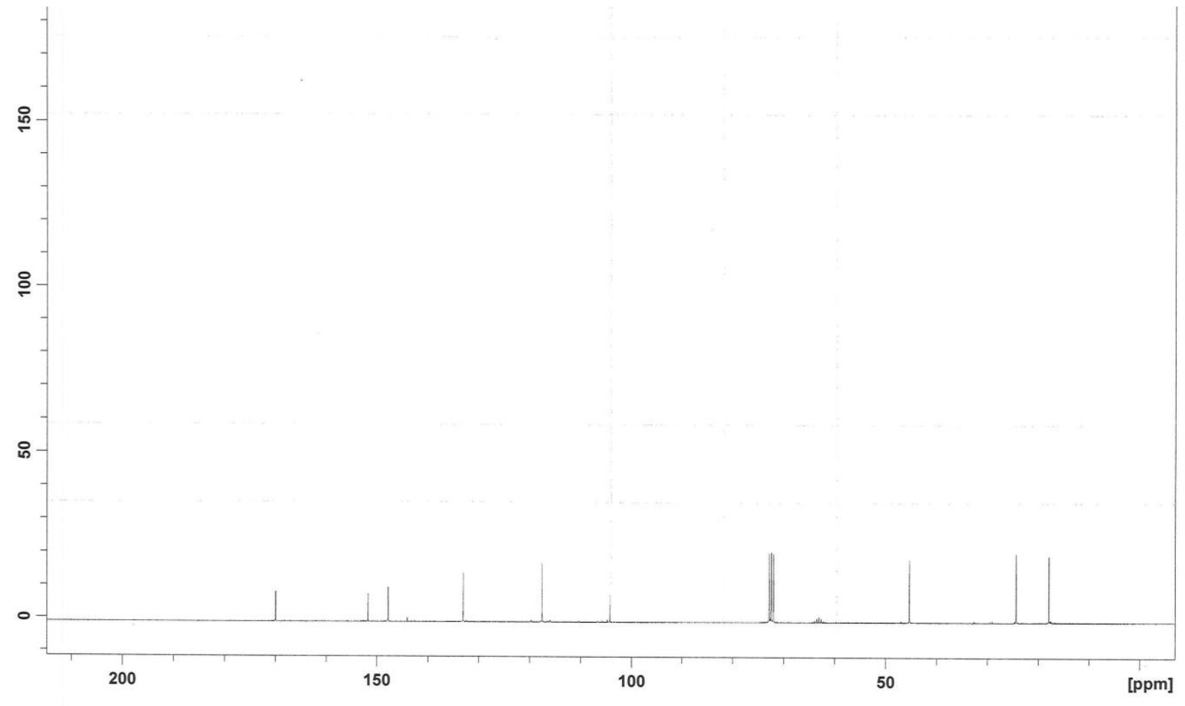

^{1}H NMR spectrum of **18**

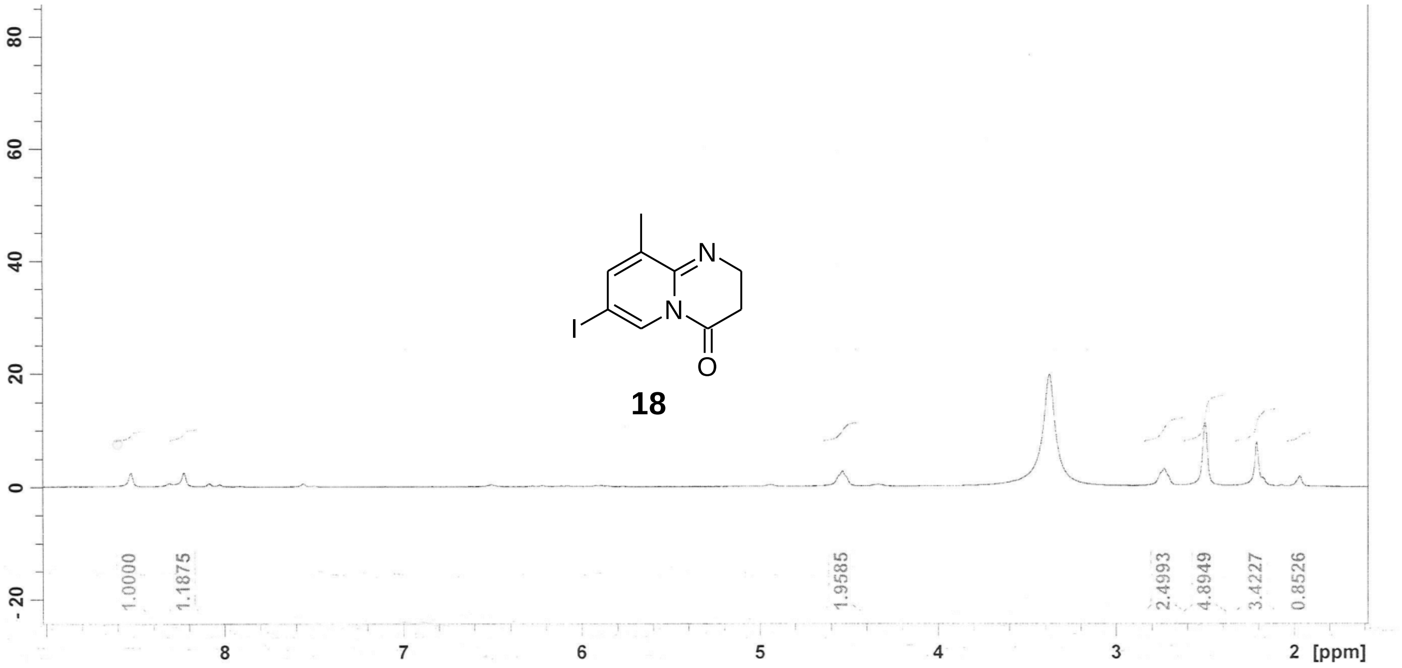

^{13}C NMR spectrum of **18**

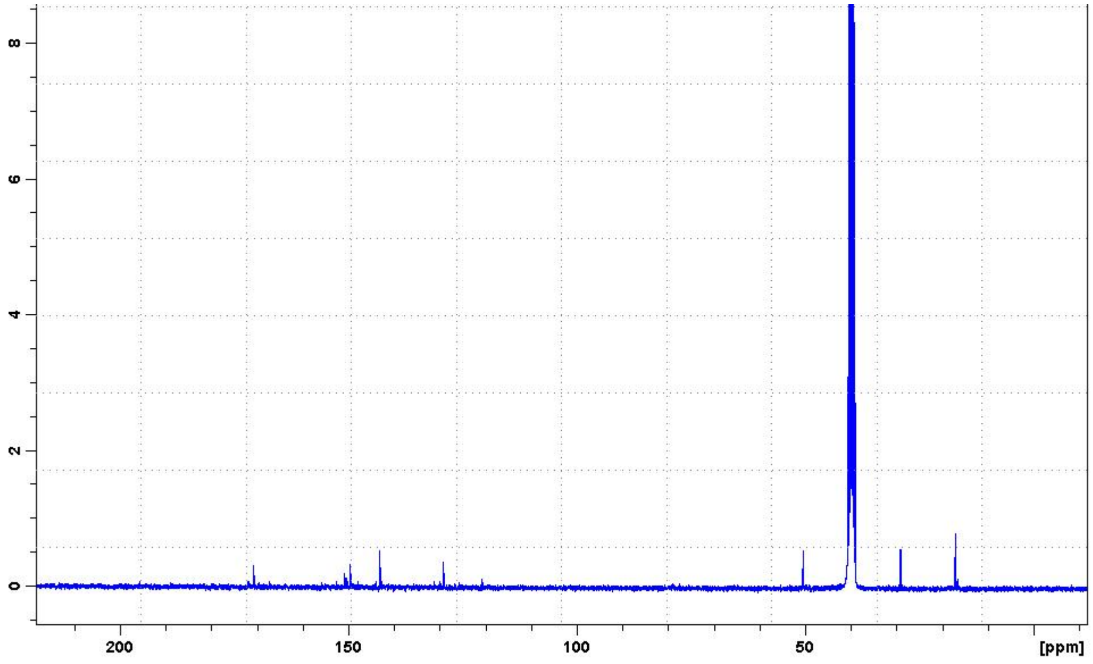

^{1}H NMR spectrum of **19**

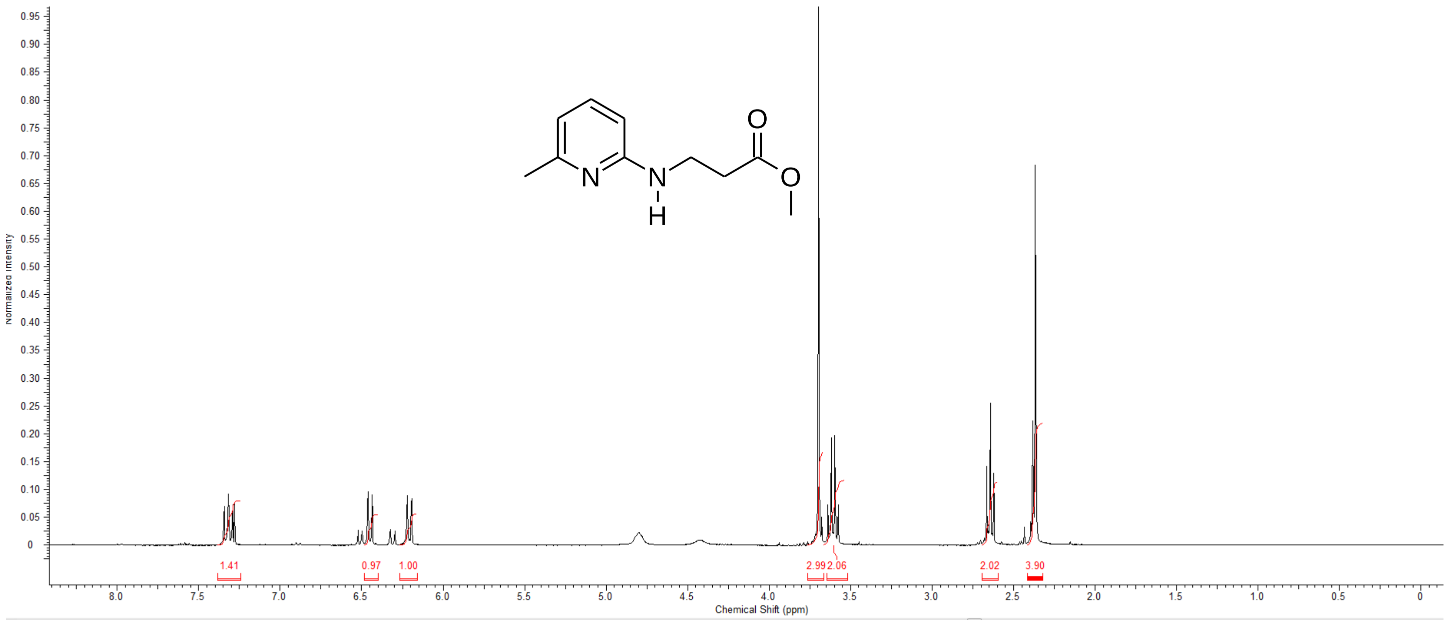

^{13}C NMR spectrum of **19**

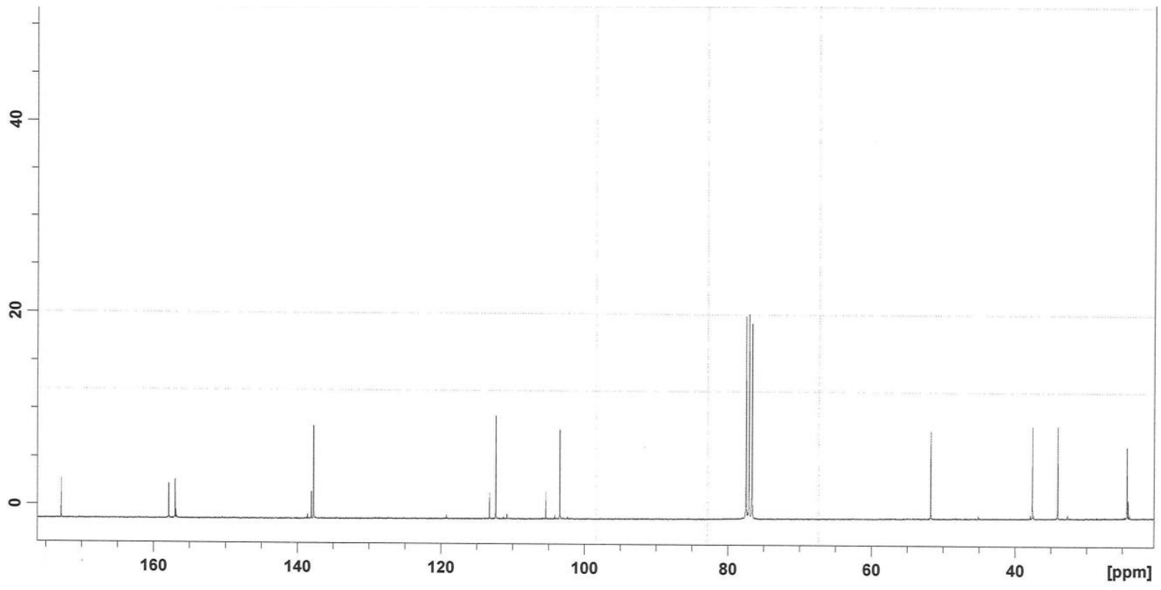

^{1}H NMR spectrum of **20**

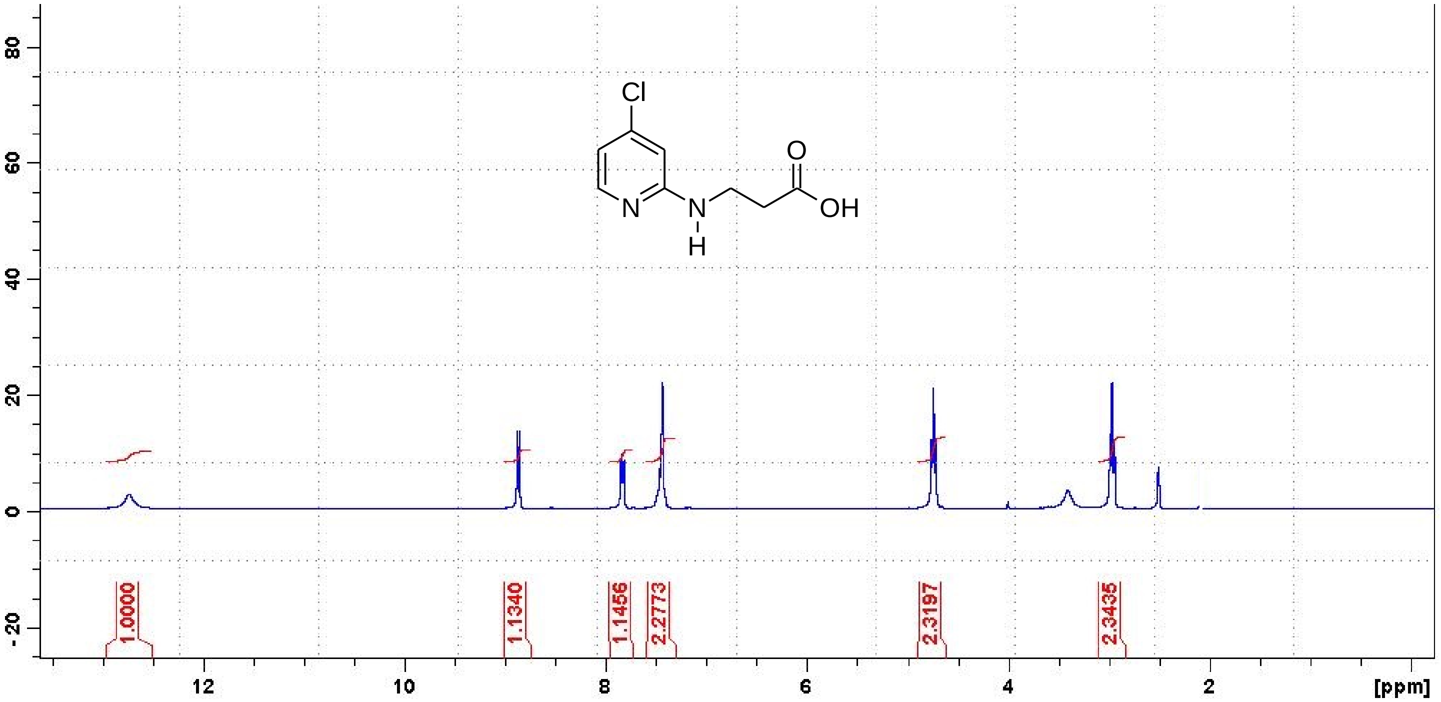

^{13}C NMR spectrum of **20**

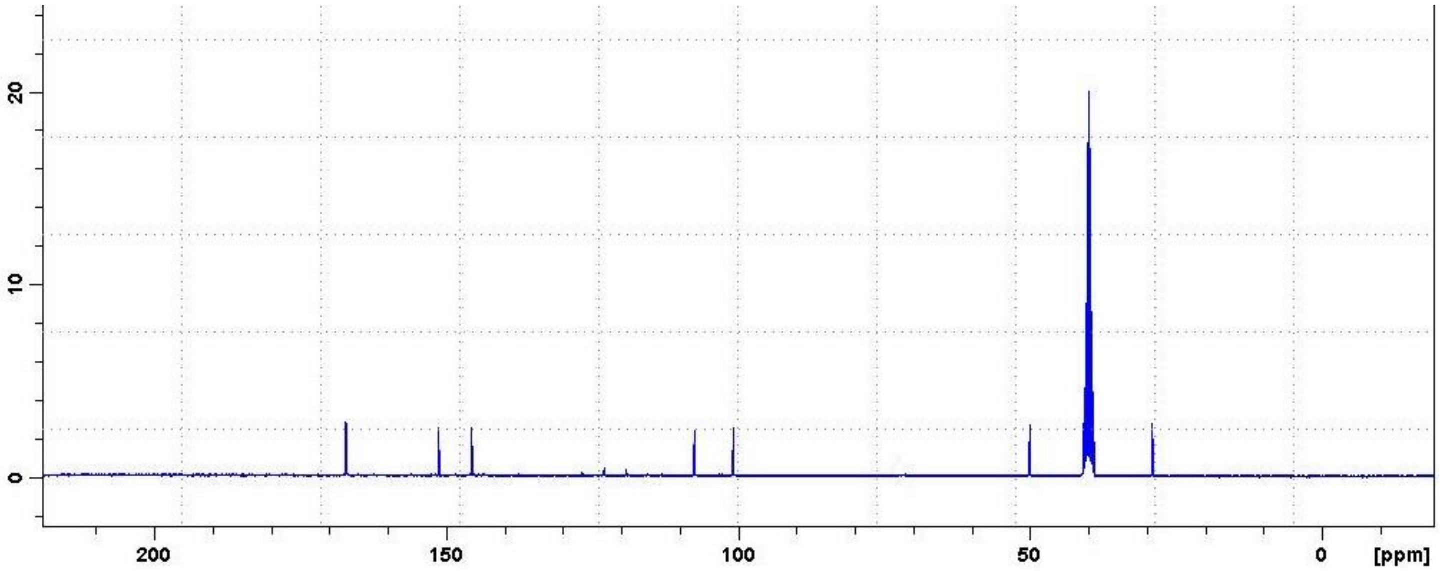

Supplement: Supplementary Information [file srep36316-s1.pdf]
